# Protein Syndesmos is a novel RNA-binding protein that regulates primary cilia formation

**DOI:** 10.1093/nar/gky873

**Published:** 2018-09-27

**Authors:** Rosario Avolio, Aino I Järvelin, Shabaz Mohammed, Ilenia Agliarulo, Valentina Condelli, Pietro Zoppoli, Giovanni Calice, Daniela Sarnataro, Elias Bechara, Gian G Tartaglia, Matteo Landriscina, Alfredo Castello, Franca Esposito, Danilo S Matassa

**Affiliations:** 1 Department of Molecular Medicine and Medical Biotechnology, University of Naples Federico II, Via S. Pansini 5, 80131 Napoli, Italy; 2 Department of Biochemistry, University of Oxford, South Parks Road, Oxford OX1 3QU, UK; 3 Proteomics Technology Development and Application, Department of Biochemistry, University of Oxford, South Parks Road, Oxford OX1 3QU, UK; 4 Laboratory of Pre-clinical and Translational Research, IRCCS, Referral Cancer Center of Basilicata, 85028 Rionero in Vulture, Italy; 5 Ceinge-Biotecnologie avanzate, s.c.a r.l., Via G. Salvatore 486, 80145, Napoli, Italy; 6 Centre for Genomic Regulation (CRG), Dr. Aiguader St. 88, 08003 Barcelona, Spain; 7 Medical Oncology Unit, Department of Medical and Surgical Sciences, University of Foggia, Viale Pinto 1, 7100 Foggia, Italy

## Abstract

Syndesmos (SDOS) is a functionally poorly characterized protein that directly interacts with p53 binding protein 1 (53BP1) and regulates its recruitment to chromatin. We show here that SDOS interacts with another important cancer-linked protein, the chaperone TRAP1, associates with actively translating polyribosomes and represses translation. Moreover, we demonstrate that SDOS directly binds RNA in living cells. Combining individual gene expression profiling, nucleotide crosslinking and immunoprecipitation (iCLIP), and ribosome profiling, we discover several crucial pathways regulated post-transcriptionally by SDOS. Among them, we identify a small subset of mRNAs responsible for the biogenesis of primary cilium that have been linked to developmental and degenerative diseases, known as ciliopathies, and cancer. We discover that SDOS binds and regulates the translation of several of these mRNAs, controlling cilia development.

## INTRODUCTION

Protein Syndesmos (SDOS, also called NUDT16L1 and TIRR) is a paralog of the nuclear decapping enzyme NUDT16. Having likely emerged by gene duplication, SDOS diverged from its ancestor by losing its decapping activity through critical sequence alterations within the catalytic NUDIX domain. In particular, a repeated glycine/leucine sequence replaced the glutamic acid residues required for catalytic activity of the NUDIX domain (1). Initially reported to promote the assembly of focal adhesions and actin stress fibers through its interaction with Syndecan 4, Paxillin and its homolog Hic-5 (2,3), it has recently been demonstrated that SDOS is a critical regulator of the double strand break (DSB) repair pathway (4). In absence of DNA damage, SDOS hijacks the component of the DSB machinery 53BP1. Upon DSB, 53BP1 is phosphorylated by the ataxia telangiectasia-mutated (ATM) kinase, promoting the interaction with Rap1-interacting factor 1 homolog (RIF-1) and the release of SDOS. In absence of SDOS, 53BP1 is recruited to chromatin (4). Overexpression of SDOS hampers the recruitment of 53BP1 to DSBs, whereas its silencing stimulates the DNA-damage-dependent association of 53BP1 with RIF-1 or PTIP (4).

In addition to its nuclear localization, SDOS has been reported to be present in the cytoplasm (2,3), although its role in this subcellular location is unknown. Our data indicate that SDOS interacts with the molecular chaperone TRAP1, suggesting a functional interplay between these two proteins. TRAP1 is a mainly mitochondrial member of the HSP90 family, whose roles in cancer cell metabolism have been extensively characterized (5,6). Interestingly, TRAP1 also localizes to the outer side of the endoplasmic reticulum (ER) membrane (7), and co-sediments with actively translating ribosomes, invoking potential roles in mRNA translation (8). TRAP1 silencing enhances the incorporation of radiolabeled amino acids and sensitizes cells to drugs targeting protein synthesis (8). Furthermore, loss of TRAP1 increases the levels of active polysomes and induces the phosphorylation of translation initiation factors (9) through the activation of the upstream AKT/p70S6K signaling pathway (10). Herein, we report that SDOS is a novel RNA-binding protein (RBP) that interacts with TRAP1 at the ER. SDOS regulates mRNA translation, and controls a group of mRNAs crucial for the biogenesis of the primary cilium. Importantly, perturbation of SDOS affects cilia formation.

## MATERIALS AND METHODS

### Cell culture

Human HCT116 colon carcinoma cells and human cervical carcinoma HeLa cells were purchased from American Type Culture Collection (ATCC) and cultured in McCoy’s 5A medium (HCT116) and Dulbecco’s modified Eagle’s medium (HeLa), supplemented with 10% fetal bovine serum and 1.5 mmol/l glutamine. The authenticity of the cell lines was verified at the beginning of the project by short tandem repeat (STR) profiling, in accordance with ATCC product description. Generation of the HeLa Flp In TRex stable cell lines expressing the eGFP-fusion proteins or the short hairpin RNA was performed according to manufacturer’s protocol (Flp In TRex, Invitrogen) and cultured in the presence of appropriate selective antibiotics. Addition of tetracycline (1 μg/ml) induces proteins as described in (11).

### Plasmid generation and transfection procedures

Full-length SDOS-myc cloned into pcDNA 3.1 myc-his vector was obtained as previously described for TRAP1-myc (7). To generate TRAP1-eGFP, SDOS-eGFP and NUDT16-eGFP plasmids, HeLa cDNA library and eGFP plasmid were used as templates for fusion polymerase chain reaction (PCR). Resulting chimeric cDNAs were cloned into pCDNA5/FRT/TO. TRAP1-Flag-HA and SDOS-Flag-HA plasmids were obtained in the same way by using the Flag-HA-tagged vector. Inducible shRNAs were generated as described in (12) (using BglII/KpnI as restriction sites in pFRT-U6tetO vector). Short hairpin sequences used are: GFP=agatctGCACAAGCTGGAGTACAACTACCTGACCCATAGTTGTACTCCAGCTTGTGCTTTTTggtacc; SDOS=agatctGCCTCAGGATGCTCTTGTTTATCCTGACCCAATAAACAAGAGCATCCTGAGGCTTTTTggtacc; TRAP1=agatctGCCCGGTCCCTGTACTCAGAAACCTGACCCATTTCTGAGTACAGGGACCGGGCTTTTTggtacc.

Transient transfection of DNA plasmids was performed with the Polyfect Transfection Reagent (Qiagen: 301105) according to the manufacturer’s protocol. TRAP1 and SDOS transient silencing were performed with siRNAs purchased from Qiagen (TRAP1: cat. no. SI00115150; SDOS: cat. no. SI00713293). For control experiments, cells were transfected with a similar amount of scrambled siRNA (Qiagen; cat. no. SI03650318). Transient transfections of siRNAs were performed using HiPerFect Transfection Reagent (Qiagen: 301704) according to the manufacturer’s protocol.

### Protein–protein interaction identification by MS

1 × 15 cm plate of TRAP1-eGFP, SDOS-eGFP and unfused eGFP expressing cells was induced for 24 h with 1 μg/ml of doxycycline. Cells were then lysed with 1 ml of lysis buffer (NaCl 150 mM, Tris–Hcl pH7.5 10 mM, Triton X-100 1%, MgCl2 5 mM, Dithiothreitol (DTT) 5 mM (fresh) and AEBSF 1× (fresh)) on ice for 15 min. Lysates were cleared by centrifugation at 16 000 × *g* for 5 min. Lysates were precleared by incubation with 50 μl of equilibrated control agarose beads (Thermo Scientific) for 30 min at 4°C under gentle rotation. eGFP-fusion proteins were then captured from precleared lysates by incubation with 40 μl of GFP-Trap agarose beads (GFP-Trap_A, Chromotek) per milliliter of lysate for 2 h, 4°C, gentle rotation. Beads were collected by centrifugation and washed six times with lysis buffer. During the second wash, samples were incubated at 37°C for 5 min with 20 μg of RNase A (Sigma R4642). Samples were eluted from the beads by pH elution as indicated in the manufacturer’s protocol (GFP-trap_A; Chromotek).

Samples were prepared using the filter aided sample preparation. Briefly, Vivacon 500 filters (Sartorius, VN01H02 10 kDa/VNCT01) were pre-washed with 200 μl 0.1% trifluoroacetic acid in 50% acetone. Samples were loaded to the filter and denatured with 200 μl 8 M urea in 100 mM triethylammonium bicarbonate buffer (TEAB) for 30 min at room temperature. Denatured proteins were reduced by 10 mM tris (2-carboxyethyl) phosphine (TCEP) for 30 min at room temperature and alkylated with 50 mM chloroacetamide for 30 min at room temperature in the dark. Subsequently, 1 μg LysC (Wako) in 150 μl 50 mM TEAB containing 6 M urea was added and incubated at 37°C for 4 h. Then the buffer was diluted to 2 M urea by 50 mM TEAB, followed by adding 0.5 μg trypsin (Promega) overnight at 37°C. Trypsinized samples were centrifuged and the flow-through containing peptides was dried and re-suspended in 70 μl 10% formic acid. Peptides were resuspended in 5% formic acid and 5% Dimethyl sulfoxide (DMSO) and then trapped on a C18 PepMap100 pre-column (300 μm i.d. × 5 mm, 100 Å, Thermo Fisher Scientific) using 0.1% formic acid in water at a pressure of 500 bar and analyzed on an Ultimate 3000 UHPLC system (Thermo Fischer Scientific) coupled to a QExactive mass spectrometer (Thermo Fischer Scientific). The peptides were separated on an in-house packed analytical column (50 cm × 75 μm i.d. packed with ReproSil-Pur 120 C18-AQ, 1.9 μm, 120 Å) and then electrosprayed directly into an QExactive mass spectrometer (Thermo Fischer Scientific) through an EASY-Spray nano-electrospray ion source (Thermo Fischer Scientific) using a linear gradient (length: 60 min, 7% to 28% solvent B (0.1% Formic acid in acetonitrile), flow rate: 200 nl/min). The raw data were acquired on the mass spectrometer in a data-dependent mode (DDA). Full scan MS spectra were acquired in the Orbitrap (scan range 350–2000 m/z, resolution 70000, AGC target 3xe6, maximum injection time 100 ms). After the MS scans, the 20 most intense peaks were selected for high-energy collision dissociation (HCD) fragmentation at 30% of normalized collision energy. HCD spectra were also acquired in the Orbitrap (resolution 17 500, AGC target 5xe4, maximum injection time 120 ms) with first fixed mass at 180 m/z. The raw data files generated were processed using MaxQuant (Version 1.5.0.35), integrated with the Andromeda search engine as previously described. To identify protein groups, peak lists were searched against mouse database as well as list of common contaminants by Andromeda. Trypsin with a maximum number of missed cleavages of two was chosen. Acetylation (Protein N-term, i.e. only the n-terminus of the protein), Oxidation (M) and Phosphorylation (S, T and Y) were used as variable modifications while Carbamidomethylation (C) was set as a fixed modification. Protein and post-translational modification (PTM) false discovery rate were set at 0.01. Match between runs was applied.

In order to compare the sets of samples, the maxquant output proteingroup text file was imported into the perseus software package (Version 1.5.5.3) and the ‘Intensity’ for each protein in all samples were subjected to a log2 transformation. The statistical significance of protein enrichment was assessed by a two-sided Student’s *t*-test within the Perseus software package. Volcano plots were obtained by plotting the log transformed *P*-value (-log10, Student’s *t*-test) against the average fold change (log2).

### Western blot and immunoprecipitation

Equal amounts of protein from cell lysates was subjected to sodium dodecyl sulphate-polyacrylamide gel electrophoresis (SDS-PAGE) and transferred to a PVDF membrane (Millipore). Protein immunoprecipitations were carried out as previously described (9). eGFP-fusion proteins were immunoprecipitated with GFP-trap magnetic agarose beads (GFP-trap_MA, Chromotek) according to manufacturer's instructions. Where indicated, protein levels were quantified by densitometric analysis using the software ImageJ (13) using βActin as the internal control and assuming the protein levels of the control equal 1. The following antibodies were used for western blotting (WB), immunofluorescence and immunoprecipitation: anti-TRAP1 (sc-13557), anti-β-ACTIN (sc-69879), anti-GAPDH (sc-69778), anti-BiP (sc-1051), anti VDAC1 (sc-8828), anti-F1ATPase (sc-16690), anti-eGFP (sc-81045), anti-MYC (sc-40), anti-VINCULIN (sc-73614), anti-PARP1 (sc-25780), anti-eIF2α (sc-133132) from SantaCruz Biotechnology; anti-SDOS (HPA044186), anti-FLAG (FT425), anti-TMEM107 (HPA052555) from Sigma-Aldrich; anti-phosphoS6 ribosomal protein (#2215); anti-eIF4E (#2067), anti-eIF4B (#3592), anti-phospho eIF2α (#3597) form Cell Signaling; anti-KIF7 (GTX130782) from Genetex; anti-rpS28 (A305-095A) form Bethyl laboratories; anti-rpL3 was kindly provided by Prof. Giulia Russo, Department of Pharmacy, University of Napoli ‘Federico II’.

### Duolink *in situ* proximity ligation assay

Duolink *in situ* proximity ligation assay (Sigma-Aldrich—DUO92101) was performed according to the manufacturer’s instructions. Briefly, cells were seeded on coverslips, fixed, permeabilized and hybridized with primary antibodies. Cells were hybridized with secondary antibodies conjugated with the PLA probes (PLUS and MINUS), and then subjected to ligation and rolling circle amplification using fluorescently labeled oligonucleotides. Cells were washed and mounted on slides using a mounting media with DAPI to detect nuclei and signal was detected by confocal microscopy analysis.

### Cell fractionation

Mitochondria and ER were purified by using the Qproteome Mitochondria Isolation kit (Qiagen—37612) according to the manufacturer’s protocol. For the collection of ribosomal and non-ribosomal fractions, the lysates were centrifuged at 10 000 × *g* at 4°C for 15 min in order to remove the mitochondria and cell debris. The supernatant was layered over a sucrose (20% wt/vol) cushion containing cycloheximide and centrifuged at 149 000 × *g* for 2 h. The pellet containing ribosomes and the upper and lower pellets of the non-ribosomal supernatants were collected. The ribosomal pellets were resuspended in the lysis buffer, after which immunoblotting was performed. Nuclear fractions were purified according to the manufacturer’s protocol (Abcam).

### Polysome profiling

3 × 10 cm plates of cells were incubated either 15 min at 37°C with fresh medium supplemented with 100 μg/ml of cycloheximide (Sigma) or 5 min at 37°C with fresh medium supplemented with 100 µg/ml of puromycin (Sigma). Cells were then washed with ice cold phosphate-buffered saline (PBS) supplemented with 100 μg/ml cycloheximide and resuspended in 1 ml lysis buffer (10 mM Tris–HCl pH 7.4, 100 mM KCl, 10 mM MgCl_2_, 1% Triton-X100, 2 U/ml Turbo DNase (Ambion), 2 mM DTT, 10 U/ml Ribolock (Invitrogen), 100 μg/ml of cycloheximide). Glass beads (Sigma-Aldrich; G8772) were added to the lysate and cells were broken by vortexing at medium speed for 3 pulses of 10 s. After 5 min of incubation on ice, cell lysate was centrifuged for 5 min at 5000 rpm at 4°C. The supernatant was collected, and the absorbance was measured at 260 nm with the NanoDrop. Eight A260 units were loaded onto a 10–50% sucrose gradient obtained by adding 6 ml of 10% sucrose over a layer of 6 ml 50% sucrose prepared in lysis buffer without Triton and containing 0.5 mM DTT, in a 12-ml tube (Polyallomer; Beckman Coulter). Gradients were obtained with the help of a gradient maker (Gradient Master, Biocomp). Polysomes were separated by centrifugation at 35 000 rpm for 3 h using a Beckmann SW41 rotor. Eleven fractions of 1 ml were collected while polysomes were monitored by following the absorbance at 254 nm. Total protein was retrieved by 100% ethanol precipitation performed overnight and analyzed by SDS-PAGE followed by western blot.

### ^35S^ Met/^35^S Cys labeling

HeLa eGFP and HeLa SDOS-eGFP cells seeded in a 6-well plate were induced for 24 h and HeLa sh-GFP and sh-SDOS for 48 h with 1 μg/ml tetracycline. HCT116 were transfected with an SDOS-directed siRNA. For control experiments, cells were transfected with a similar amount of non-targeting control siRNA. Following proteins induction or silencing, cells were incubated in cysteine/methionine-free medium (Sigma-Aldrich) for 15 min followed by incubation in cysteine/methionine-free medium containing 50 μCi/ml ^35^S-labeled cysteine/methionine (Perkin-Elmer) for 30 min. Cells were then washed with PBS and lysed. A total of 10 μg of total protein extract was analyzed by SDS-PAGE and autoradiography.

### PNK assay

Cells expressing eGFP-fusion proteins were UV-crosslinked on ice (150 mJ/cm^2^), lysed (100 mM KCl; 5 mM MgCl_2_, 10 mM Tris pH 7.5, 0.5% NP40; 1 mM DTT; protease inhibitor cocktail) and homogenized passing the lysate through a narrow needle (22G) followed by pulsed ultrasonication (3 × 10 s, 50% amplitude, on ice). Cleared lysates were treated with 50 U/ml DNAseI (Takara) and RNaseI for 15 min at 37°C, and used for immunoprecipitation with GFP-Trap_A agarose beads (Chromotek) for 2 h at 4°C. Beads were washed four times with High salt buffer (500 mM NaCl, 20 mM Tris pH 7.5, 1 mM MgCl_2_, 0.05% NP40, 0.1% SDS, complete) and two times with PNK buffer (50 mM Tris pH 7.4, 50 mM NaCl, 10 mM MgCl_2_, 0.5% NP40, 5 mM DTT). RNA crosslinked to the tagged RBP is identified by radiolabeling with 0.1 μCi/μl γ-32P ATP by T4 polynucleotide kinase (1 U/μl) in PNK buffer (50 mM NaCl, 50 mM Tris pH 7.5, 0.5% NP40, 10 mM MgCl_2_ and 5 mM DTT) for 15 min at 850 rpm and 37°C. Beads were washed four to six times with PNK buffer and protein–RNA complexes were eluted by boiling samples 5 min at 95°C. Samples were analyzed by SDS-PAGE and autoradiography. For Flag-HA fusion proteins expressing cells the protocol described in (14) was followed.

### Interactome capture for eGFP-tagged proteins

1 × 15 cm plate of eGFP-fusion protein expressing cells was induced for 24 h (TRAP1) and 16 h (SDOS, NUDT16 and eGFP) with 1 μg/ml doxycycline. TRAP1-eGFP and SDOS-eGFP cells were treated with 100 μM 4-thiouridine overnight and photoactivatable ribonucleoside-enhanced crosslinked (PAR-CL) on ice at 0.60 and 0.30 J cm^−2^ with UV light at 365 nm. Following UV-irradiation the protocol was performed as previously described (15).

### Individual nucleotide crosslinking and immunoprecipitation (iCLIP)-seq

1 × 15 cm plate of eGFP-fusion protein expressing cells was induced for 16 h with 1 μg/ml doxycycline. Cells were treated with 100 μM 4-thiouridine overnight and photoactivable ribonucleoside-crosslinked on ice at 0.60 and 0.30 J cm^−2^ with UV light at 365 nM. Immediately after irradiation, cells were lysed in 1 ml of lysis buffer (NaCl 100 mM, MgCl_2_ 5mM, Tris pH 7.5 10 mM, NP40 0.5%, SDS 0.1%, Na deoxycholate 0.5%, DTT 1 mM (fresh), 1× AEBSF (fresh). The cell lysate was passed three times through 27 1/2G needle and sonicated using a bioruptor (Digenode) for three cycles of 10 s (pause 15 s), level M at 4°C, then it was cleared by centrifugation at 17 900 *g* for 10 min at 4°C. RNA was then partially digested by adding 10 μl of 1:100 dilution of RNase I (Ambion, AM2295), as well as 2 μl of Turbo DNase (Ambion, AM 2238). After 3 min of incubation at 37°C under shaking at 1100 rpm 11 μl of Ribolock (Invitrogen) were added to each lysate. The lysates were precleared by incubation with 50 μl of equilibrated control agarose beads (Thermo Scientific) for 30 min at 4°C under gentle rotation. eGFP-fusion proteins were then captured from precleared lysates by incubation with 40 μl of GFP-Trap agarose beads (GFP-Trap_A, Chromotek) per ml of lysate for 2 h, 4°C, gentle rotation. Beads were collected by centrifugation and washed twice with High salt buffer (NaCl 500 mM, Tris–HCl pH7.5 20 mM, MgCl_2_ 1 mM, NP40 0.05%, SDS 0.10%, 1× AEBSF (fresh)); twice with Medium salt buffer (NaCl 250 mM, Tris–HCl pH7.5 20 mM, MgCl_2_ 1 mM, NP40 0.05%, 1× AEBSF (fresh)) and twice with Low salt buffer (NaCl 150 mM, Tris–HCl pH 7.5 20 mM, MgCl_2_ 1 mM, NP40 0.01%, 1× AEBSF (fresh)). The RNA was dephosphorylated, and 3′-linker ligated as described in (16). The protein/RNA complexes were isolated as described in (17). Samples were processed for subsequent steps as described in (16). cDNA libraries obtained after PCR amplification with universal Solexa primers (25 cycles) were multiplexed and sequenced using an Illumina Next-generation sequencing platform at Science for Life Laboratory at Karolinska Insitue, Solna, Sweden.

The data is publicly available at the Gene Expression Omnibus (GEO) database under ID GSE118050.

### Gene expression profile assay

Total RNA from SDOS-eGFP and control eGFP HeLa cells was extracted using TRIzol reagent (Invitrogen) after 24 h of induction with Tetracycline (1 μg/ml).

RNA concentration was evaluated with a NanoDrop 2000c spectrophotometer (Thermo Scientific), its quality was assessed with a Bioanalyzer 2100 (Agilent Technologies). For each sample, 300 ng of total RNA were reverse transcribed and used for synthesis of cDNA and biotinylated cRNA according to the Illumina TotalPrep RNA amplification kit protocol (Ambion). A total of 750 ng of each cRNA were hybridizated on Illumina HumanHT12 v4.0 Expression BeadChip array (Illumina); staining was performed according to standard protocol supplied by Illumina. BeadChip was dried and scanned with an Illumina HiScanSQ system (Illumina). Analysis was performed in triplicate for each sample.

The data is publicly available at the Gene Expression Omnibus (GEO) database under ID GSE118050.

### RNA extraction and RT-qPCR analysis

Total RNA extraction was performed by using the TRI Reagent (Sigma-Aldrich, product code T9424) following the manufacturer’s instruction. For first-strand synthesis of cDNA, 1 μg of RNA was used in a 20-μl reaction mixture by using a SensiFast cDNA synthesis kit (Bioline). For real-time PCR analysis, 0.4 μl of cDNA sample was amplified by using the SensiFast Syber (Bioline) in an iCycler iQ Real-Time Detection System (Bio-Rad Laboratories GmbH, Segrate, Italy).

The reaction conditions were 95°C for 2 min followed by 40 cycles of 5 s at 95°C and 30 s at 60°C. Actin was used as internal control. The full list of primers used is reported in Supplementary Materials and Methods.

### Ribosome profiling

Ribosome profiling was performed according to the protocol described in (18). Briefly, unfused eGFP and SDOS-eGFP cells were cultured in 15 cm plates and induced with doxycycline for 24 h. After 15 min incubation with 100 μg/ml cycloheximide (Sigma-Aldrich; C4859) at 37°C, cells were washed with ice cold PBS and 1 ml of lysis buffer (20 mM Tris–HCl pH 7.4, 150 mM NaCl, 5 mM MgCl_2_, 1 mM DTT, 100 μg/ml cycloheximide, 1% Triton-X100) was added. Cells were then collected and incubated on ice; glass beads (Sigma-Aldrich; G8772) were added to the lysate and cells were broken by vortexing at medium speed for 3 pulses of 10 s. After 10 min of incubation on ice, lysates were centrifuged for 10 min at 10 000 rpm at 4°C and the supernatant was recovered. RNA was partially digested with 3.5 μl of RNase I (100 U/μl, Invitrogen AM2294) per 800 μl of lysate. After 15 min of incubation at 24°C, lysates were placed on ice and supplemented with 10 μl of SUPERaseIn (20 U/μl, Invitrogen AM2694). Lysates were then loaded on a 34% sucrose cushion (34% sucrose in 20 mM Tris–HCl pH 7.4, 150 mM NaCl, 5 mM MgCl_2_, 1 mM DTT and 100 μg/ml cycloheximide) and monosomes were pelleted by centrifugation for 1 h at 70 000 rpm using a Beckman TLA 100.3 rotor. RNA was extracted from the pellet and ribosome-protected fragments (RPFs) of 30 nt were purified as described (18). RPFs were depleted of ribosomal RNA with the Ribo-Zero rRNA removal kit (Epicentre MRZH116) according to manufacturer’s indications. cDNA libraries were generated according to (18) and sequenced by Solexa using a HiSeq 2000, Single Read, 50 nt at the CRG Genomics Core Facility, Barcelona, Spain.

The data is publicly available at the Gene Expression Omnibus (GEO) database under ID GSE118050.

### GFP-trap immunoprecipitation and RT-qPCR

1 × 15 cm plate of SDOS-eGFP and eGFP control cells was induced for 16 h with 1 μg/ml doxycycline. SDOS-eGFP cells were treated with 100 μM 4-thiouridine overnight and photoactivable ribonucleoside-crosslinked on ice at 0.30 J cm^−2^ with UV light at 365 nM. eGFP cells were crosslinked on ice at 0.15 J cm^−2^ with UV light at 254 nM. Immediately after irradiation, cells were lysed in 1 ml of lysis buffer (NaCl 100 mM, MgCl2 5 mM, Tris pH 7.5 10 mM, NP40 0.5%, SDS 0.1%, Na deoxycholate 0.5%, DTT 1 mM (fresh), 1× AEBSF (fresh), 100 U/ml Ribolock RNase inhibitor, 200 μM ribonucleoside vanadyl complex). The cell lysate was passed three times through 27 1/2G needle and sonicated using a bioruptor (Digenode) for 3 cycles of 10 s (pause 15 s), level M at 4°C, then it was cleared by centrifugation at 17 900 *g* for 10 min at 4°C. A total of 50 μl of input were used to measure fluorescence signal at plate reader in order to normalize the amount of eGFP proteins to be immunoprecipitated. A total of 30 μl of control magnetic agarose beads (Pierce) and GFP-trap_MA beads (Chromotek) were equilibrated in Dilution buffer (NaCl 500mM, MgCl_2_ 1 mM, SDS 0.05%, NP40 0.05%, Tris pH 7.5 50mM, 100 U/ml Ribolock RNase inhibitor (fresh), 1× AEBSF (fresh)). Lysates were pre-cleared for 30 min under rotation at 4°C with 30 μl of control magnetic agarose beads. GFP-trap_MA were incubated with *Escherichia coli* tRNA (1 mg/ml) for 15 min in dilution buffer under rotation at 4°C and then washed two times with dilution buffer. Pre-cleared lysates were then incubated with GFP-trap_MA beads for 2 h under rotation at 4°C. Beads were then washed two times with High salt buffer (NaCl 500 mM, Tris pH 7.5 20 mM, MgCl_2_ 1 mM, NP40 0.05%, SDS 0.1%, Ribolock RNase inhibitor 100 U/ml (fresh), 1× AEBSF (fresh) and three times with Low salt buffer (NaCl 150 mM, Tris pH 7.5 20 mM, MgCl_2_ 1 mM, NP40 0.01%, Ribolock RNase inhibitor 50 U/ml). Beads were resuspended in 100 μl of Proteinase K buffer (NaCl 0.1M, Tris pH 7.5 10 mM, ethylenediaminetetraacetic acid 1 mM, SDS 0.5%, 200 μg/ml Proteinase K, 50 pg spike-in control RNA) and incubated at 55°C for 1 h under constant mixing. To recover RNA, 100 μl of TRI Reagent were directly added to the buffer-containing beads followed by extraction and ethanol precipitation. The RNA was reverse transcribed, and the resulting cDNA was analyzed by quantitative PCR. The amount of precipitated RNA from immunoprecipitates (IPs) was normalized to the amount of the spike-in control.

### Confocal microscopy and analysis of the cilium

HeLa cells were seeded on coverslips, induced for 24 h (GFP/SDOS-GFP) or 48 h (shGFP/shSDOS), then serum-starved for 48 h to induce the formation of the cilium. Cells were then fixed with 4% (w/v) paraformaldehyde in PBS for 20 min, blocked and permeabilized with 0.4% (w/v) BSA, 0.1% (v/v) Triton X-100, 5% (v/v) FBS in PBS for 15 min at RT before staining over night with alpha Tubulin (acetyl K40) antibody (ab179484, Abcam, Cambridge, UK). Cells were then analyzed with a Leica TCS-SMD Sp5 confocal microscope by using Argon laser (488 nm) and DPSS laser (561 nm). Single-cell analysis of cilia length (expressed in micron) was carried out by using LAS AF software on maximal projection of at least 20 optical Z-slices of 0.5-micron-thick acquired with a 63 × 1.4 NA immersion oil objective.

### Bioinformatic analyses

Bioinformatic analyses of gene expression profiling, iCLIP and Ribosome profiling, Gene Ontology (GO) are reported in Supplementary Materials and Methods.

## RESULTS

### SDOS and TRAP1 are protein partners

**Figure 1.**
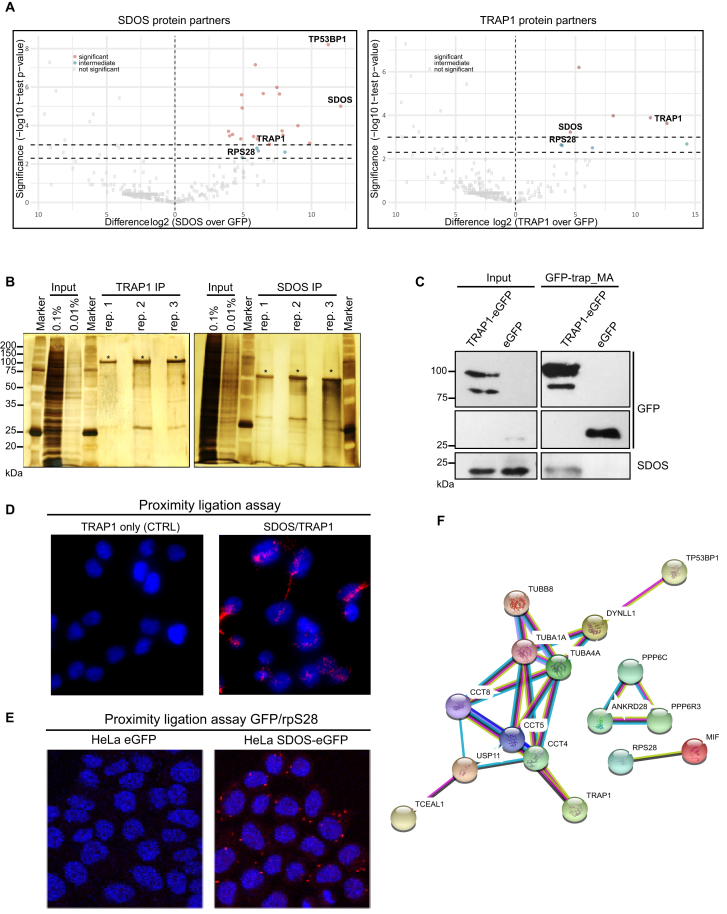
SDOS and TRAP1 are protein partners. (**A**) Volcano plot showing SDOS (left) and TRAP1 (right) protein partners identified by MS (dark-red = significant, light blue = intermediate, light gray = not significant). Relevant protein-partners (RPS28, SDOS, TRAP1, TP53BP1) are highlighted. (**B**) Silver staining showing the pattern of SDOS-eGFP and TRAP1-eGFP IPs (specific band indicated with asterisk). (**C**) Co-immunoprecipitation of TRAP1 and SDOS from HeLa cells following induction of TRAP1-eGFP and eGFP for 24 h. Total lysates were immunoprecipitated with GFP-Trap_MA magnetic agarose beads and analyzed by WB with the indicated antibodies. (**D**) Representative image of PLA showing the interaction of SDOS with TRAP1 in HCT116 cells. Positive signals of interaction are shown as red dots, nuclei are stained with DAPI (blue). Negative control has been obtained by hybridizing cells with TRAP1 antibody only. (**E**) Representative image of PLA showing the interaction of SDOS with rpS28 in HeLa cells, following induction of SDOS-eGFP or unfused eGFP (negative control) for 24 h and hybridization with anti-GFP and anti-rpS28 antibodies. Positive signals of interaction are shown as red dots, nuclei are stained with DAPI (blue). (**F**) Computational prediction of SDOS protein partners network using STRING database (https://string-db.org). Interactions include direct (physical) and indirect (functional) associations. Light-blue edge: known interaction from curated databases; purple edge: experimentally determined interaction; green edge: textmining; black edge: co-expression; dark-blue edge: protein homology.

Preliminary results from our lab showed that TRAP1 interacts with the poorly characterized protein SDOS. To confirm and functionally characterize the potential interaction between SDOS and TRAP1, we generated HeLa cell lines expressing either SDOS-eGFP or TRAP1-eGFP in an inducible manner. eGFP fusion proteins were immunoprecipitated with the highly specific nanobody against eGFP (19) in presence of RNases and the eluates were analyzed by label-free quantitative proteomics (Figure 1A). The purity of SDOS and TRAP1 IPs was confirmed by silver staining (Figure 1B). Mass spectrometry revealed several SDOS and TRAP1 interactors that were absent in the unfused eGFP control (Tables 1 and 2). These protein partners are expected to bind directly to SDOS and TRAP1 as the RNase treatment would disrupt any interaction mediated by RNA. The quality of our proteomic results was highlighted by the identification of 53BP1 as the primary interactor of SDOS, as previously described (4). Strikingly, SDOS and TRAP1 were strongly enriched in both SDOS-eGFP and TRAP1-eGFP immunoprecipitations, demonstrating that they are high confidence interaction partners (Figure 1A). This interaction was further confirmed by co-immunoprecipitation by isolating TRAP1-eGFP and revealing endogenous SDOS, which is totally absent in the unfused eGFP IP (Figure 1C). We further visualized TRAP1/SDOS interaction by *in situ* proximity ligation assay (PLA) in HCT116 colorectal carcinoma cells. Using specific antibodies against SDOS and TRAP1, PLA revealed specific stained foci not only confirming their direct interaction in living cells, but also indicating that such interaction may occur in the cytoplasm (Figure 1D).

**Table 1.** SDOS interacting proteins obtained by label-free quantitative proteomics

Gene names	Protein names	Significance (log10 *t*-test *P*-value)	Difference (SDOS/eGFP log2 FC)
TP53BP1	Tumor suppressor p53-binding protein 1	8.25077	11.0722
CCT5	T-complex protein 1 subunit epsilon	7.1619	5.88357
DYNLL1; DYNLL2	Dynein light chain 1, cytoplasmic;Dynein light chain 2, cytoplasmic	5.98194	7.46163
SLC7A5	Large neutral amino acids transporter small subunit 1	5.66293	6.46204
CCT4	T-complex protein 1 subunit delta	5.64192	7.6541
SLC16A3	Monocarboxylate transporter 4	5.59991	4.88154
TUBA4A	Tubulin alpha-4A chain	4.91233	4.91643
TUBB8	Tubulin beta-8 chain	3.99008	9.00189
TUBA1A; TUBA3C; TUBA3E	Tubulin alpha-1A chain;Tubulin alpha-3C/D chain;Tubulin alpha-3E chain	3.72152	7.84447
PPP6C	Serine/threonine-protein phosphatase 6 catalytic subunit	3.70021	3.92096
POLDIP2	Polymerase delta-interacting protein 2	3.53627	4.22019
SQSTM1	Sequestosome-1	3.4972	7.90479
ANKRD28	Serine/threonine-protein phosphatase 6 regulatory ankyrin repeat subunit A	3.46259	4.00495
MIF	Macrophage migration inhibitory factor	3.43102	5.75599
BAG2	BAG family molecular chaperone regulator 2	3.30518	6.02968
PPP6R3	Serine/threonine-protein phosphatase 6 regulatory subunit 3	3.30512	4.82287
LGALS3BP	Galectin-3-binding protein	3.08069	9.86244
TRAP1	Heat shock protein 75 kDa, mitochondrial	3.035	6.92604
TCEAL1	Transcription elongation factor A protein-like 1	2.82362	6.00085
HNRNPH3	Heterogeneous nuclear ribonucleoprotein H3	2.67817	6.1067
NUDT16	U8 snoRNA-decapping enzyme	2.61619	8.05465
RPS28	40S ribosomal protein S28	2.3294	4.91507
CCT8	T-complex protein 1 subunit theta	2.25142	6.83071
SLC25A10	Mitochondrial dicarboxylate carrier	2.20143	5.34728
USP11	Ubiquitin carboxyl-terminal hydrolase 11	2.17424	7.29486
LAMA1	Laminin subunit alpha-1	2.10802	4.40167
SLC25A6; SLC25A4	ADP/ATP translocase 3;ADP/ATP translocase 1	1.98901	5.70873
HDAC6	Histone deacetylase 6	1.84778	3.31599
SBSN	Suprabasin	1.82849	4.65037
CPS1	Carbamoyl-phosphate synthase [ammonia], mitochondrial	1.64454	5.035
CCT2	T-complex protein 1 subunit beta	1.63926	4.41436
CCT7	T-complex protein 1 subunit eta	1.54531	5.05293
S100A7; S100A7A	Protein S100-A7;Protein S100-A7A	1.53909	5.10047
HAGH	Hydroxyacylglutathione hydrolase, mitochondrial	1.39384	2.8948
HSPA8	Heat shock cognate 71 kDa protein	1.37934	6.06043
TOMM40	Mitochondrial import receptor subunit TOM40 homolog	1.32786	3.5971
SNRPA1	U2 small nuclear ribonucleoprotein A	1.30897	5.05506
HSPA5	78 kDa glucose-regulated protein	1.30267	5.11503

**Table 2.** TRAP1 interacting proteins obtained by label-free quantitative proteomics

Gene names	Protein names	Significance (log10 *t*-test *P*-value)	Difference (SDOS/eGFP log2 FC)
ABCF2	ATP-binding cassette sub-family F member 2	2.68385	14.2995
TOMM40	Mitochondrial import receptor subunit TOM40 homolog	3.88582	11.2559
ACAT1	Acetyl-CoA acetyltransferase, mitochondrial	3.97718	8.15674
HSP90AB1	Heat shock protein HSP 90-beta	2.50603	6.42103
CPS1	Carbamoyl-phosphate synthase [ammonia], mitochondrial	1.90015	6.23542
ACTA1; ACTC1; ACTG2; ACTA2	Actin, alpha skeletal muscle;Actin, alpha cardiac muscle 1;Actin, gamma-enteric smooth muscle;Actin, aortic smooth muscle	1.71572	6.06877
HIST1H1B	Histone H1.5	1.94157	5.88322
MIF	Macrophage migration inhibitory factor	6.19998	5.29286
SNRPA1	U2 small nuclear ribonucleoprotein A	1.34302	5.17618
SBSN	Suprabasin	1.93546	5.10971
NUDT16L1	Protein syndesmos	3.22837	4.57307
S100A7; S100A7A	Protein S100-A7;Protein S100-A7A	1.31531	4.29323
POLDIP2	Polymerase delta-interacting protein 2	2.61116	3.89041
RPS28	40S ribosomal protein S28	2.64818	3.7948
GHITM	Growth hormone-inducible transmembrane protein	1.65522	3.29815

To determine whether SDOS–TRAP1 complex contains other proteins, we searched in our proteomic dataset for interactors common for both proteins (Tables 1 and 2). Interestingly, the ribosomal protein S28 (RPS28) was the only protein present in both SDOS and TRAP1 immunoprecipitations, suggesting that SDOS may associate with ribosomes as previously shown for TRAP1 (8,9). The interaction between SDOS and rpS28 was validated by PLA (Figure 1E). Out of the 39 interactors identified, 12 (31%) (TUBA1A, CCT2, CCT4, CCT5, CCT7, CCT8, SLC25A4, HSPA5, HSPA8, PPP6R3, ANKRD28, HDAC6) are present in the recently published mammalian ribo-interactome (20). In addition, STRING network analysis of SDOS partners (21) highlighted an enrichment in proteins involved in protein folding and *de novo* posttranslational protein folding such as TRAP1, CCT4, CCT5, CCT8, TUBA1A, TUBA4A (Figure 1F). The CCT complex associates co- and post-translationally with approximately 5–10% of the newly made proteins (22), aligning well with the hypothesis of SDOS binding to ribosomes.

### SDOS associates with actively translating polyribosomes

**Figure 2.**
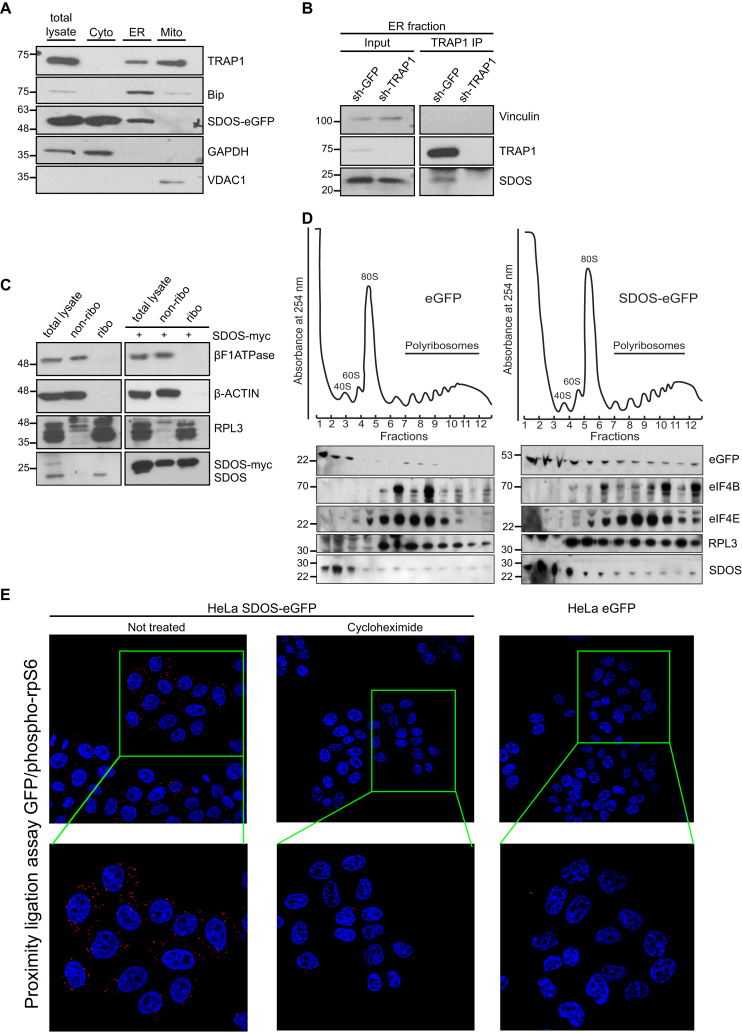
SDOS localizes on the ER and associates with actively translating polyribosomes. (**A**) Subcellular fractionation of HeLa cells into mitochondrial (Mito), cytosolic (Cyto) and microsomal (ER) fractions following 24 h induction of SDOS-eGFP. Total lysate and fractions were analyzed by WB with indicated antibodies. BiP is used as a ER marker, GAPDH as a cytosolic marker and VDAC1 as a mitochondrial marker. (**B**) WB analysis of TRAP1-IP from the ER fraction with SDOS, TRAP1 antibodies. Vinculin is used as internal control. (**C**) Ribosomal purification from HCT116 cells and HCT116 cells transfected with SDOS-myc, followed by immunoblot with β-ACTIN (as a cytosolic marker), βF1ATPase (as a mitochondrial marker), RPL3 (as a ribosomal marker) and SDOS antibodies. (**D**) Polysome profiling absorbance, measured at 254 nm, indicates the sedimentation of the particles: fractions 1 and 2 free cytosolic proteins or light complexes; fractions from 3 to 7 ribosomal subunits (60S, 40S) and monomer (80S); fractions from 8 to 12 polysomes. Proteins from each fraction were analyzed by WB with the indicated antibodies. rpL3 was used as a ribosome marker, eIF4B and eIF4E were used as markers of translation initiation complex. (**E**) Representative image of PLA showing the interaction of SDOS-eGFP with phosphorylated (active) ribosomal protein S6 (phospho-rpS6) in HeLa cells following 24 h induction of SDOS-eGFP by using GFP and phospho-rpS6 antibodies. Positive signals of interaction are shown as red dots, nuclei are stained with DAPI (blue). Cycloheximide treatment (200 μg/ml, 1 h) was used to confirm the causal role of active protein synthesis on the binding.

SDOS was first identified as a cytosolic protein involved in the assembly of focal adhesions and actin stress fibres (2,3). However, it has recently been shown that this protein also localizes in the nucleus—like its paralog NUDT16p—where it interacts with 53BP1 (4). In agreement with both studies, we confirmed that SDOS localizes in both nucleus and cytoplasm (Supplementary Figure S1A).To get more insights into the cytoplasmic localization of SDOS, we performed cell fractionation experiments. WB analysis of sub-cellular fractions of HeLa SDOS-eGFP revealed that a proportion SDOS is present at the ER (Figure 2A). In agreement, endogenous SDOS was also identified in the ER fraction (Supplementary Figure S1B). In agreement with previous data (7), beside the ‘canonical’ mitochondrial localization, TRAP1 was found in the ER fraction (Figure 2A). Since the distribution of both proteins only overlaps at the ER, it is likely that the interaction between these two proteins occurs at this compartment. To confirm this possibility, we performed a TRAP1 IP from the ER fraction and observed that SDOS is co-isolated with TRAP1, suggesting that both proteins interact at the ER (Figure 2B). Importantly, SDOS was not immunoprecipitated when TRAP1 was depleted with shRNAs, demonstrating that the antibody against TRAP1 does not cross-react with SDOS (Figure 2B).

SDOS localization at the ER and its interaction with the ribosomal protein RPS28 (Table 1) suggest that it may associate with ribosomes, in analogy to its protein partner TRAP1 (8,9). To test this hypothesis, we isolated ribosomal fraction from HCT116 total cell extracts. Analysis of the ribosomal (ribo) and non-ribosomal (non-ribo) fractions by WB demonstrated that both the endogenous SDOS and ectopically expressed myc-tagged SDOS associate with ribosomes (Figure 2C). To verify if the association of SDOS with ribosomes might reflect a functional role in protein synthesis, we performed a polysome profiling analysis in SDOS-eGFP and unfused eGFP HeLa cells. Both endogenous SDOS and ectopically expressed SDOS-eGFP co-sediment with actively translating polyribosomes, whereas unfused eGFP failed to do so (Figure 2D). Notably, association of SDOS with ribosomes is prevented by inhibiting protein synthesis with puromycin, which causes disassembly of active 80S ribosomes (Supplementary Figure S1C). Accordingly, a PLA using antibodies against phospho(active)-ribosomal protein S6 and eGFP upon expression of SDOS-eGFP HeLa cells showed intense staining at multiple cytoplasmic foci. These foci were virtually absent in the eGFP control but also dramatically reduced in the SDOS-eGFP cells upon translation arrest triggered by 1 h treatment with cycloheximide (200 μg/ml) (Figure 2E). In agreement, inhibition of protein synthesis by emetine or puromycin also yields significant reduction in proximity ligation when using antibodies against SDOS and p-rpS6 (Supplementary Figure S1D). Shorter (15 min) treatment with cycloheximide 100 μg/ml, which is the concentration and incubation time typically used in polysome profiling to prevent ribosomal run-off, did not disrupt the interaction between SDOS and p-rpS6 (Supplementary Figure S1D). This milder treatment did not induce eIF2α phosphorylation, which is a marker of translation initiation arrest, opposite to emetine or the stronger cycloheximide treatment (Supplementary Figure S1E). These data thus confirm that SDOS is present in the ribosomal fraction and that this localization is dependent on active protein synthesis.

### SDOS affects protein synthesis

**Figure 3.**
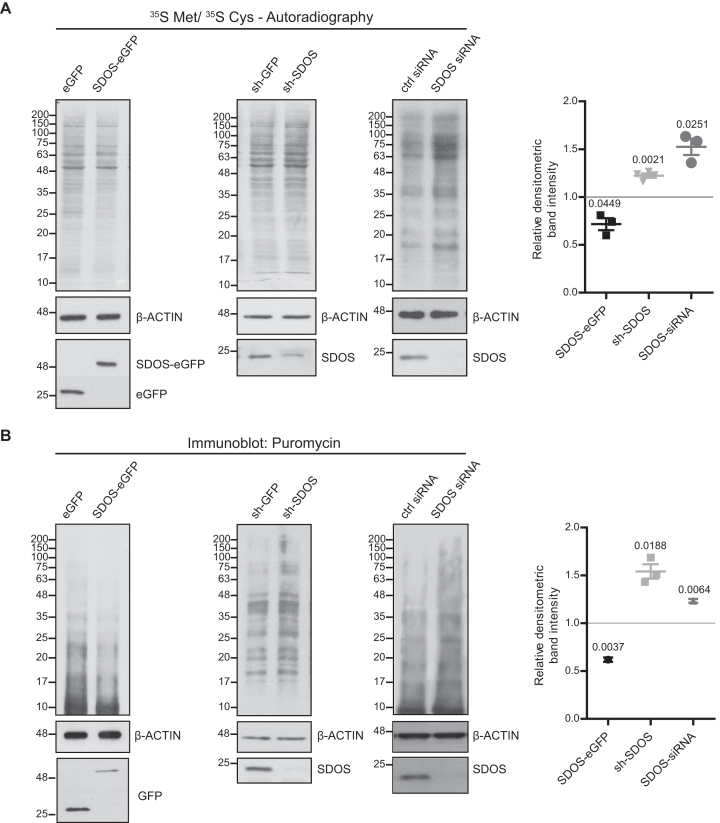
SDOS overexpression represses translation of specific mRNAs. (**A**) Autoradiography of total lysates from cells labeled with ^35^S Met/^35^S Cys (left) and relative densitometric band intensities (right), calculated by assuming protein levels of the control equal 1, following 24 h induction of eGFP/SDOS-eGFP or 48 h of shGFP/shSDOS in Hela FITR or transfection of SDOS-targeting siRNA and non-targeting control siRNA in HCT116 cells. The *P*-value in the graph indicate the statistical significance based on one-sample *t*-test (*n* = 3). (**B**) WB analysis of total lysates following puromycin treatment (1 μg/ml, 15 min) (left) and relative densitometric band intensities (right), calculated by assuming protein levels of the control equal 1. The *P*-value in the graph indicate the statistical significance based on one-sample *t*-test (*n* = 3).

To verify if SDOS plays an active role in the regulation of translation, we assessed protein synthesis by radioactive labeling with [^35^S]-Met/Cys followed by autoradiography, either upon overexpression or silencing of SDOS. SDOS-eGFP cells incorporate less radiolabeled amino acids into newly synthesized proteins than the cells expressing unfused eGFP (Figure 3A). Conversely, the lack of SDOS caused the opposite result, promoting higher rates of [^35^S]-Met/Cys incorporation in both HeLa and HCT116 cells to a similar extent (Figure 3A). Taking together, these data indicate that SDOS attenuates protein synthesis. To support these results, we treated cells with puromycin, which binds to nascent peptides and detected these newly synthesized polypeptides by WB with antibodies against puromycin. This approach yielded similar outcome to the [^35^S]-Met/Cys incorporation; i.e. overexpression of SDOS repressed translation, whereas SDOS silencing increased it (Figure 3B). These data indicate that SDOS influences translation, possibly though a direct interaction with ribosomes.

### SDOS is a novel RNA-binding protein

**Figure 4.**
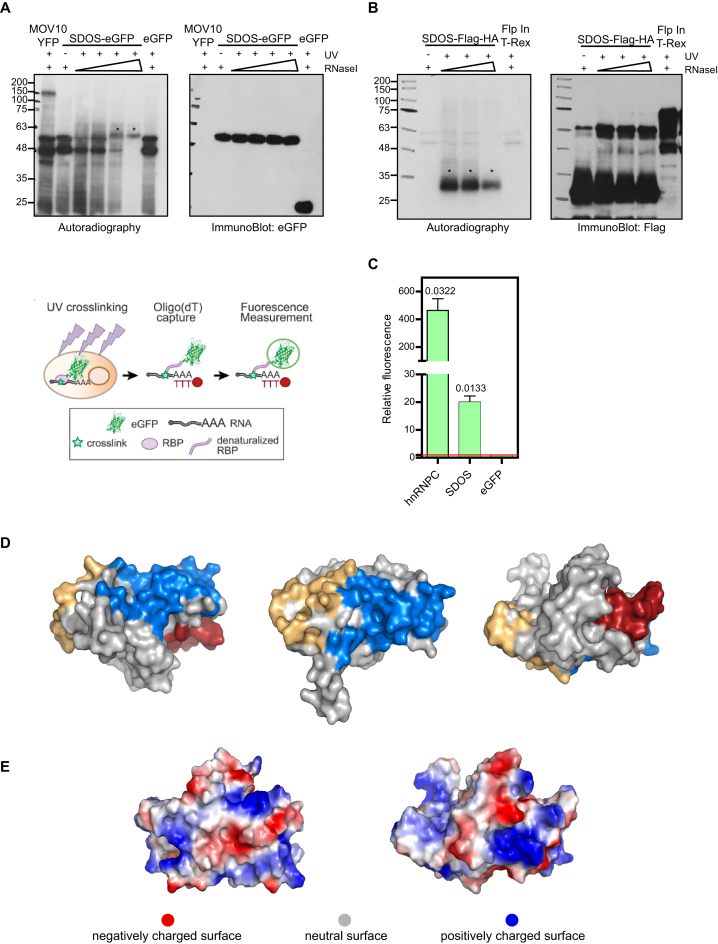
SDOS is a RBP. (**A** and **B**) Autoradiography and WB analysis of eGFP-fused (A) and Flag-HA tagged (B) proteins after PNK assay in the presence of increasing concentration of Rnase I (panel A: 1, 10, 50, 100 ng/µl; panel B: 1, 10, 100 ng/µl). MOV10-YFP was used as positive control. Unfused eGFP and M2-Flag beads were used as negative control, respectively. Asterisks on autoradiography indicate specific bands. (**C**) eGFP-based RNA-binding assay with schematic representation of the procedure (left) and relative green fluorescence signal of RNA-bound fraction over input from cells expressing different eGFP fusion proteins (right). Numbers above bars indicate the statistical significance (*P*-value), based on one-sample *t*-test (*n* = 3). (**D**) BindUP RNA-binding surface prediction of SDOS (PDB ID: 3kvh). The most likely RNA-binding surfaces are represented in blue and orange, while the NUDIX domain is represented in red. (**E**) Electrostatic surface potential of SDOS. The protein surface is represented according to charge.

SDOS harbors a catalytically inactive NUDIX domain, but it may retain RNA-binding activity (1). To evaluate whether SDOS interacts with RNA in living cells, we performed crosslinking and immunoprecipitation (CLIP) followed by T4 polynucleotide kinase (PNK) assay. In brief, cells are irradiated with UV light to promote protein–RNA crosslinks, and then RNA is trimmed by RNase treatment and the protein–RNA complex is immunoprecipitated. The PNK treatment incorporates a [^32^P] to the 5′ of the RNA remnant crosslinked to the protein. Bands matching the molecular weight of SDOS-eGFP and the positive control MOV10-eGFP were detected by autoradiography in an UV and RNase-dependent manner, while no signal was observed in the eGFP immunoprecipitation (Figure 4A). This indicates that SDOS interacts with RNAs in living cells. Notably, the same results were obtained with a different tag (SDOS-Flag-HA) (Figure 4B). Considering that SDOS is a paralog of the RNA decapping enzyme NUDT16, it is conceivable that SDOS RNA-binding capacity could be underestimated if SDOS binds to the 5′ cap structure, which cannot be phosphorylated by PNK. To further validate SDOS RNA-binding activity with an orthogonal approach, we used RNA-interactome capture (RNA-IC) (11,23). In brief, RNA-IC also employs UV crosslinking of cultured cells expressing SDOS-eGFP recombinant protein, but in this case the RBP is isolated by hybridization of the poly(A) tail present in the target RNA with oligo(dT) magnetic beads, via stringent washes. The presence of the eGFP-fusion protein in the eluates is detected by fluorescence using a plate reader (Figure 4C) (14). Importantly, we detected significant green fluorescence in eluates from the cells expressing SDOS-eGFP as well as the positive control hnRNPC-eGFP, a well-known canonical RBP, while no signal was observed in the negative control (eGFP) (Figure 4C). It is important to mention that hnRNPC crosslinks with RNA between 10 and 100 times more efficiently than the majority of well-established RBPs (11,14,15). By using these two complementary approaches, we demonstrate that SDOS binds RNA in living cells, thus providing the first evidence that SDOS is a novel RBP. To predict the most likely RNA-binding surface within SDOS, we used BindUP software (24), which calculates the local electrostatic potential to predict protein surfaces compatible with RNA binding (Figure 4D and E). BindUP identified two large positively charged patches as candidates for mediating the interaction with RNA (Figure 4D, blue and orange). Interestingly, the region compatible with RNA binding in SDOS is also present in NUDT16 (Supplementary Figure S2A and B). As a consequence RNA-binding activity of NUDT16 and SDOS to RNA, measured by RNA-IC, is comparable (Supplementary Figure S2C). The amino acids forming the patches predicted by BindUP are listed in Supplementary Table S1.

### Identification of the RNAs bound by SDOS

**Figure 5.**
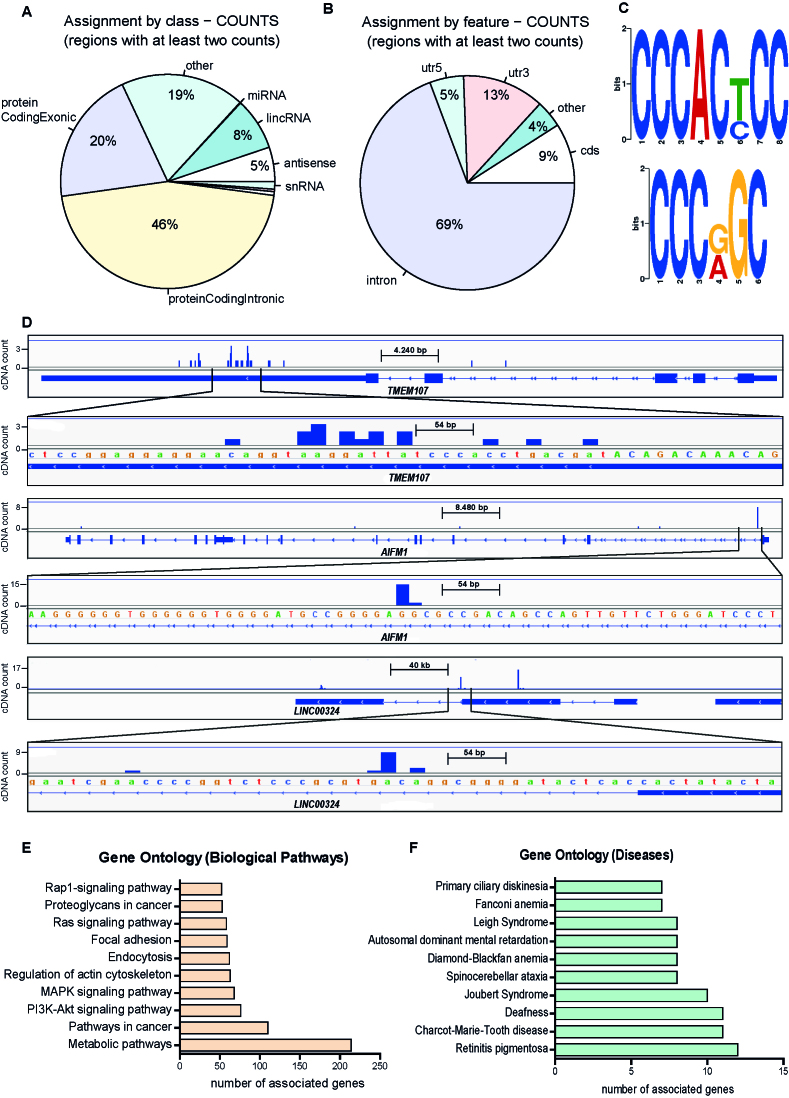
SDOS binds RNAs in multiple regions. (**A** and **B**) Genomic location of nucleotides crosslinked to SDOS. Pie charts depicts assignment by class (A) or feature (B) of RNA bound, as given on the right. (**C**) Logos for two enriched sequences around ±12 bp of 448 strongest SDOS binding sites. (**D**) Conversion of mapped iCLIP sequence reads into cDNA count values for *Tmem107, Aifm1* and *Linc00324*. (**E** and **F**) GO analysis showing the top 10 biological pathways (E) and the top 10 diseases (F), whose associated mRNAs are enriched in the iCLIP dataset.

To determine the pool of RNAs bound by SDOS, we performed individual nucleotide resolution CLIP followed by sequencing (iCLIP-seq). We obtained a set of 4456 SDOS target RNAs identified in three biological replicates (Supplementary Table S2). The vast majority (66%) of the RNAs bound by SDOS are protein-coding transcripts (mRNAs, Figure 5A), while only 8% correspond to long intergenic non-coding RNAs (lincRNAs). Most of the reads (69%) mapped to introns, but also coding regions (9%), 3′UTRs (13%) and 5′UTR (5%) (Figure 5B and D). While a decapping enzyme is expected to bind mostly to 5′ UTRs (25), the preferential binding of SDOS to other mRNA regions suggests that the sequence alterations compared to NUDT16 have profound consequences in its binding potential. Interaction with introns is likely mediated by the nuclear SDOS pool and may have consequences in RNA splicing. In agreement with this idea, our protein–protein interaction analysis revealed that SDOS interacts with HNRNPH3 (Table 1), which is localized in nuclear bodies and is involved in splicing (26). Analysis of enriched motifs sequence motifs around the strongest 10% of SDOS binding sites revealed a preference for C-rich sequences (Figure 5C and Supplementary Figure S3). SDOS has been reported to form homo-dimers (4,27,28), and adjacent C-rich motifs may reflect co-binding for two SDOS proteins to same RNA (Supplementary Figure S3).

GO analysis of the SDOS-bound mRNA network shows enrichment in pathways involved in focal adhesion and actin cytoskeleton organization, i.e. ‘Rap1-signaling pathway’, ‘focal adhesion’, ‘regulation of actin cytoskeleton’ and ‘MAPK signaling pathway’, among the top 10 enriched biological processes (Figure 5E). This agrees with earlier studies reporting a direct role of SDOS in development of focal adhesions (2,3). Notably, the top three enriched biological processes are ‘metabolic pathways’, ‘pathways in cancer’ and ‘PI3K-Akt signaling pathway’ (Figure 5E). This is in agreement with suggested role of SDOS in cancer (4,29), PI3K-Akt mediated cellular processes being among the crucial mechanisms in tumour development (30) and considering that emerging evidence support the definition of cancer as a metabolic disease (31). Remarkably, some SDOS RNA targets are linked to neurological disorders and ciliopathies such as Joubert syndrome, Retinitis pigmentosa, Primary ciliary dyskinesia (Figure 5F). Ciliopathies are a group of developmental and degenerative diseases that affect virtually all organs and tissues and are commonly caused by defects in primary cilia formation or function (32). Therefore, SDOS may be involved in the expression of numerous relevant genes with direct links to disease.

### Identification of transcripts translationally regulated by SDOS

**Figure 6.**
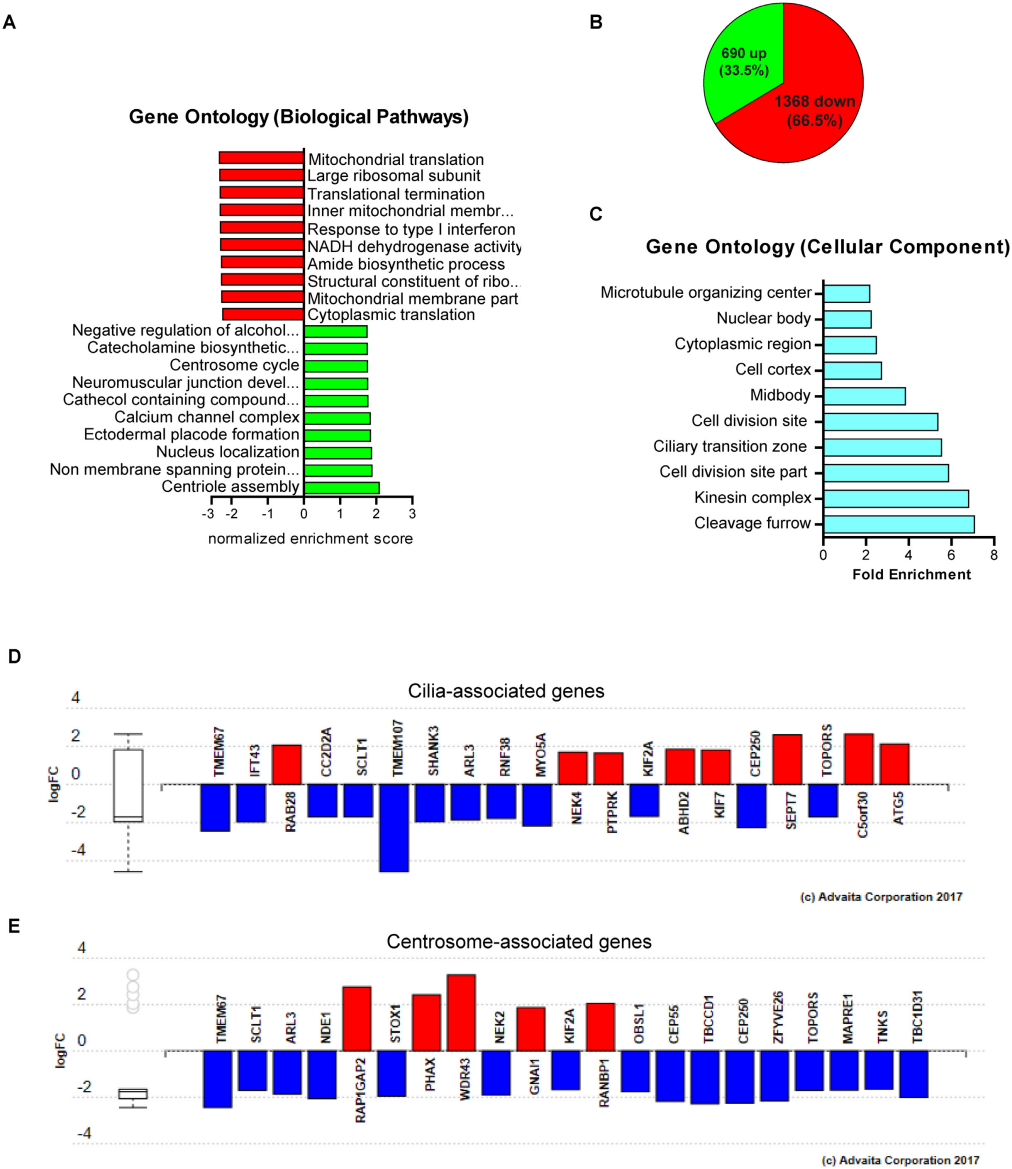
SDOS iCLIP and RP show common transcripts. (**A**) GO analysis showing the top 10 over- (green) and under- (red) represented significant biological pathways associated to GE data. (**B**) Pie chart representing the ratio between up- and downregulated mRNAs according to RP-Seq analysis following SDOS-eGFP overexpression. (**C**) GO analysis showing the top 10 significantly enriched cellular component terms associated to targets common to both iCLIP and RP data. (**D**) Ciliopathy-associated genes found among the RP and iCLIP common targets according to Advaita Bio’s iPathwayGuide analysis. (**E**) Cilia and centrosome-associated genes from RP and iCLIP common targets according to Advaita Bio’s iPathwayGuide cellular component analysis.

Different lines of evidence suggest that SDOS plays a role in translation: (i) SDOS interacts with TRAP1 and RPS28 at the ER (Figures 1 and 2), (ii) it co-sediments with ribosomes (Figure 2 and Supplementary Figure S1) perturbation of SDOS causes alterations in protein synthesis (Figures 3 and 4) SDOS is an RBP that interacts with numerous mRNAs (Figure 4). To determine whether perturbation of SDOS causes alterations in RNA levels, and if such alterations could cause the changes in protein synthesis rate detected upon SDOS up- or downregulation, we employed a microarray analysis upon SDOS-eGFP overexpression in HeLa cells (Supplementary Table S3). The quality of the microarray analysis was confirmed by RT-qPCR, showing significant regulation of 10 out of 14 mRNAs with differential expression in SDOS-eGFP detected by microarrays (Supplementary Figure S4A). GO analysis of the mRNAs with reduced abundance in cells overexpressing SDOS revealed enrichment in the GO terms ‘cytoplasmic translation’, ‘translational termination’, ‘large ribosomal subunit’, ‘structural constituent of ribosomes’ and ‘mitochondrial translation’ (Figure 6A). This indicates that SDOS could, additionally, regulate translation indirectly influencing the abundance of mRNAs encoding ribosomal proteins. On the other hand, the mRNAs with increased abundance after SDOS overexpression are enriched in GO terms ‘nucleus localized genes’ and ‘centrosome cycle’, which agrees with the existence of a nuclear population of SDOS and the discovered link between SDOS and cilia-linked genes, respectively (Figure 6A), considering that the primary cilium originates from the centrosome and is coordinated with the cell cycle (33). However, only 23 up- and 56 downregulated RNAs showed a 1.5 < fold change > 0.75, suggesting that mRNA abundance is not the main determinant of the translation attenuation caused by SDOS overexpression.

To directly identify those transcripts whose translation may be affected by SDOS, we performed ‘ribosome profiling and sequencing’ (RP-seq) after SDOS overexpression. This technique identifies the RNA regions protected by ribosomes from RNase cleavage leaving ribosome footprints (i.e. ribosome-protected fragments, RPF) in a transcriptome-wide manner (18). We identified 2058 mRNAs (ribosome profiling) (Supplementary Table S4) differentially translated in SDOS-eGFP versus eGFP cell lines. Interestingly, only 79 of these mRNAs displayed differential expression (RNA abundance) with *P* < 0.05, indicating that SDOS has little effects on RNA synthesis and stability, while it is evidently involved in translation. Out of the 2058 translationally regulated mRNAs, 690 (33.5%) were upregulated, while 1368 (66.5%) were downregulated (Figure 6B), further supporting the role played by SDOS in translation repression. Regulated mRNAs are enriched for transcripts encoding for proteins localized to midbody, chromatin, nuclear speckle, nucleolus, cytosol and Golgi apparatus. Speckles are membrane-less cellular compartment where splicing factors accumulate. Therefore, SDOS may regulate mRNA splicing directly (i.e. through direct binding to introns, as suggested by the binding to intronic regions of the transcripts and by the presence, in the proteomic analysis of protein partners of HNRNPH3, which is involved in the splicing process) or/and indirectly (i.e. through translational regulation of mRNAs encoding splicing factors).

To determine the scope of mRNAs identified by both iCLIP and ribosome profiling, which are thus bound by SDOS and regulated at a translational level, we cross-referenced the iCLIP and the ribosome profiling datasets. A subset of 399 mRNAs was present in both datasets and we considered these as ‘direct’ targets, since they are both bound by SDOS and regulated at translation (Supplementary Table S5). These transcripts were mainly translationally repressed in SDOS-eGFP expressing cells (241 out of 399 mRNAs, 60.4%). Conversely, 158 mRNAs were translationally upregulated (39.6%). GO analysis of these direct targets showed enrichment for ciliary transition zone and kinesin complex (which is involved in cilium biogenesis) among the cellular component associated terms (Figure 6C). Ciliopathy-associated genes and cilia and centrosome associated genes found in the ‘direct target’ list are reported in Figure 6D and E. Among them, *Tmem67, Cc2d2a* and *Kif7* mRNAs were linked to Joubert syndrome and Meckel Gruber syndrome, both belonging to the ciliopathy family. Mutation or dysregulation of *Cc2d2a, Tmem67, Kif7* plus *Tmem107* and *Topors*, are known to cause ciliopathies (34). Taken together, these analyses strongly suggest that SDOS is involved in the regulation of primary cilia assembly.

### SDOS interaction with mRNAs is involved in primary cilia formation

**Figure 7.**
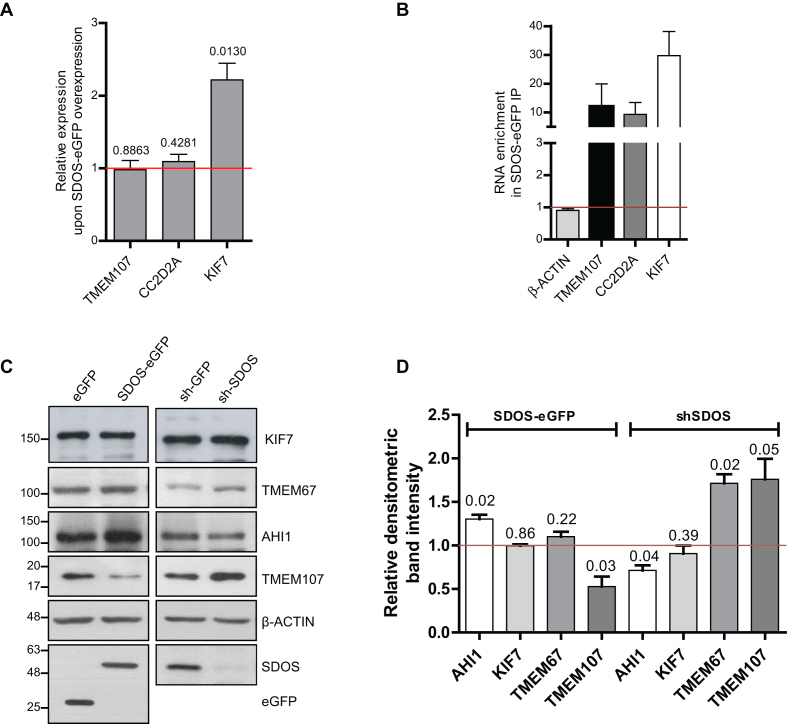
SDOS binds and regulates translation of mRNAs encoding for ciliary components. (**A**) RT-qPCR of *Cc2D2a, Kif7* and *Tmem107* mRNAs from HeLa cells upon 24 h of SDOS-eGFP or eGFP induction (βActin used as the internal control). Data are expressed as mean ± S.E.M. from four independent experiments with technical triplicate each. Numbers above bars indicate the statistical significance (*P*-value), based on one-sample *t*-test. Red line indicates expression level of the relative eGFP control. (**B**) Representative results of RT-qPCR from RIP independent experiments to validate iCLIP data. RNA enrichment was normalized to a spike-in control (red line). Actin was used as negative control showing no enrichment. (**C** and **D**) WB analysis of HeLa cell extracts following SDOS-eGFP or eGFP (24 h) and sh-GFP or sh-SDOS (48 h) induction (C), with relative densitometric analysis (D), calculated by assuming protein levels of the control equal 1. The *P*-value in the graph indicate the statistical significance based on one-sample *t*-test (*n* = 3).

We selected a subset of 11 candidate mRNAs identified by iCLIP and ribosome profiling and involved in cilia formation or/and ciliopathies, for further validation. Among those, only *Kif7* was upregulated in RT-qPCR experiments (ß-actin was used as the internal control, as neither its levels are affected by SDOS nor it is bound by SDOS) (Figure 7A). None of the other cilia-related mRNAs found in the SDOS iCLIP were affected by SDOS overexpression (Figure 7A and Supplementary Figure S4B). We then focus our attention on *Cc2d2a, Tmem107* and *Kif7 and* confirmed by RNA immunoprecipitation (RIP) that mRNAs are bound by SDOS, as they were significantly enriched in SDOS-eGFP IPs over the eGFP negative control (Figure 7B). As expected, no enrichment was observed for the control *ß-actin* mRNA, which is not a SDOS mRNA target according to the iCLIP data. Although *Kif7* mRNA levels were increased by SDOS-eGFP overexpression by nearly 2-fold (Figure 7A), it was enriched by 30-fold in the IPs, confirming that *Kif7* mRNA is also bound by SDOS (Figure 7B). Finally, we performed WB analyses to determine whether SDOS perturbation affects protein expression of cilia-linked transcripts, as indicated by ribosome profiling (Figure 7C). AHI1 and TMEM107 fully confirmed RP-seq data. Indeed, the levels of TMEM107 after SDOS-eGFP overexpression were lower than in the eGFP control, while the opposite outcome was observed for AHI1. Accordingly, both TMEM107 and AHI1 levels were inversely regulated upon SDOS silencing. Although RP-seq showed translational regulation of *Kif7* mRNA upon SDOS overexpression, we could not detect changes in protein levels by WB, which may be due to high stability or compensatory mechanisms. TMEM67 protein levels were also unaffected by SDOS overexpression; however, SDOS silencing resulted in an increased expression of TMEM67, confirming the translational impact detected with RP-seq (Figure 7D).

**Figure 8.**
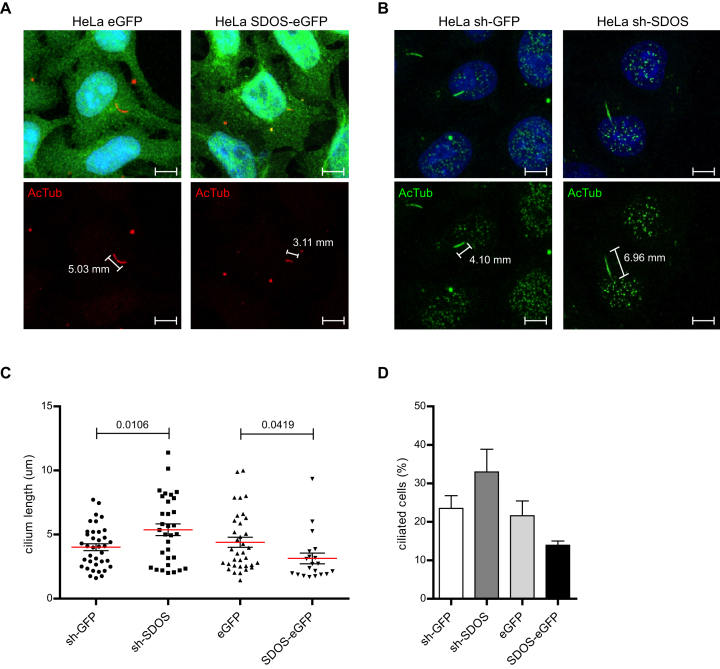
SDOS expression alters cilium formation. (**A**) Representative image of the primary cilium (red) and its length in HeLa cells upon overexpression of SDOS-eGFP or the respective eGFP control. Cilium formation has been induced by serum starvation (48 h), then cells have been fixed, permeabilized and hybridized with Acetyl-Tubulin as a primary cilium marker. Nuclei are stained with DAPI (blue). Scale bar: 15 μM. (**B**) Representative image of the primary cilium (green) and its length in HeLa cells upon induction of SDOS-directed shRNA and the respective shGFP control. Cilium formation has been induced by serum starvation (48 h), then cells have been fixed, permeabilized and hybridized with Acetyl-Tubulin as a primary cilium marker. Nuclei are stained with DAPI (blu). Scale bar: 15 μM. (**C**) Length of primary cilia measured by confocal microscopy analysis of Acetyl-Tubulin-stained HeLa cells upon SDOS overexpression (SDOS-eGFP, *n* = 146) or silencing (shSDOS, *n* = 110) and relative eGFP (*n* = 165) and shGFP (*n* = 168) controls. Numbers indicate the statistical significance (*P*-value) based on the Student’s *t*-test. (**D**) Bar graph representing the percentage of ciliated HeLa cells upon SDOS silencing (shSDOS) or overexpression (SDOS-GFP) compared to the respective shGFP and eGFP controls.

Since SDOS regulates several mRNAs involved in the biogenesis of the primary cilium, we hypothesized that perturbation of SDOS may alter cilium formation. To address this, cells overexpressing (Figure 8A) or lacking (Figure 8B) SDOS were serum deprived for 48 h to induce the formation of primary cilia. Cilia were then stained by acetylated tubulin antibodies and their length was measured by confocal microscopy analysis (Figure 8C). Although length of primary cilia is highly variable between cells and is influenced by several factors (35), we strikingly found that SDOS silencing significantly increased average cilium length, while SDOS overexpression reduced it (Figure 8C). Moreover, lack of SDOS led to a higher percentage of cilium-presenting cells than in control cells following starvation, whereas SDOS overexpression reduced it (Figure 8D). These data confirm that SDOS controls cilia formation.

## DISCUSSION

Using a multiple orthogonal methods we have shown that the poorly characterized protein SDOS is a novel RBP that regulates numerous mRNAs at the translational level and plays a central role regulating cilia formation. Our iCLIP data suggests that SDOS has a strong preference for C-rich sequences, commonly CCCA (Figure 5 and Supplementary Figure S3). Most sequence-specific interactions with RNA involve binding to the nucleotide bases (36). Since UV crosslinking is mediated by the nucleotide bases (37), SDOS-RNA crosslinking reflects direct contacts between amino acids and bases placed at ‘zero’ distances. This phenomenon is illustrated by the RBDmap experiments, where the alpha-helix of the DEAD-box helicase fold contacting the nucleotide bases crosslinks to RNA, while the domain regions interacting with the sugar-phosphate backbone failed to do so (14). Furthermore, the patch detected by BindUP in SDOS as putative RNA-binding surface does not only contain amino acids with potential to establish electrostatic interactions with the phosphate-backbone (i.e. R and K), but also aromatic (e.g. W, H, F) and hydrophilic residues (e.g. T, S, N, D, E) with capacity to stack and to form hydrogen bonds, respectively (36). Hence, SDOS has the potential to establish specific interactions with RNA, although future structural analyses are required to visualize the spatial arrangement between amino acids and ribonucleotides in these interactions. Indeed, we demonstrate that SDOS interacts with a subset of transcripts enriched in mRNAs encoding for centrosomal proteins and component of the primary cilium, and regulates their translation. Cilia are a sensory appendage that are present in most mammalian cells and plays critical roles in signaling pathways and cell-cycle progression. Mutation or dysregulation of several of these genes are linked to rare diseases called ciliopathies, a group of developmental and degenerative diseases that affect almost all organs and tissues (34). Indeed, we have found several genes crucially related to the development of various ciliopathies as mRNA bound and/or regulated at translation by SDOS. Among those, *Kif7*, a cilia-associated protein belonging to the kinesin family, whose genetic mutations have been associated with various ciliopathies; *Ahi1*, which is required for ciologenesis and whose mutations cause specific forms of Joubert syndrome; *Tmem67*, which is required for ciliary structure and function and whose genetic defects cause Meckel syndrome and Joubert syndrome; *Tmem107*, which regulates ciliogenesis and ciliary protein composition and whose mutations cause different ciliopathies, including Meckel–Gruber syndrome and orofaciodigital syndrome; *Cc2d2a*, which plays a critical role in cilia formation and whose mutations cause Meckel syndrome and Joubert syndrome (32,34).

For a long time, SDOS has been a protein with elusive functional role and poor characterization, and only recently it has been demonstrated that SDOS is a critical regulator of the DSB repair pathway (4). Initially, SDOS was reported to be involved in the regulation of cell migration, either through its binding to the cytoplasmic domain of Syndecan 4 (2) or to the focal adhesion adaptor protein Paxillin (3). Interestingly, Paxillin localizes at the base of primary cilia next to the basal body in mammalian ciliated cells (38). Syndecan itself has multiple connections with the non-canonical Wnt pathway, an ancient and evolutionarily conserved pathway that is crucial for regulation of cell fate determination, migration and polarity (39). The Wnt pathway, in turn, is dysregulated in the ciliopathies due to disruption of physiological role of the primary cilium, a pivotal transducer of extracellular signals through this pathway (40). In this context, our study provides evidence for a link between the diverse SDOS functions. Notably, besides Wnt, many of the signaling systems that control cell migration, another function previously associated to SDOS (2,3), are also coordinated by primary cilia, such as Hh, TGFβ and RTK signaling (41–44). Relevant to the connection between the newly discovered functions of SDOS in the regulation of DSB repair and cilium biogenesis, an emerging area of research claims the involvement of cilia and centrosomal protein in DNA damage response (DDR) mechanisms. Cilia, centrosomes and the DDR are linked in several ways: (i) the DDR pathway functions at the centrosome, (ii) ciliopathy proteins function in the nucleus during DDR and (iii) DDR and cilia share common regulatory proteins (45). One of these is ATMIN, a co-factor for ATM in the response to DNA damage (46) and also a transcriptional regulator of ciliary dynein, DYNLL1 (47). Intriguingly, this protein was found in our MS analysis of SDOS protein partners. Moreover, ATM is the factor responsible for the dissociation of the SDOS–53BP1 complex, necessary for the subsequent binding of 53BP1 to the effector proteins during DDR (4).

Asymmetric localization of proteins can be achieved by mRNA localization coupled to protein synthesis at a distal site (48); however, cilia have no protein synthesis machinery, therefore all protein components for the cilium needs to be synthesized within the cytosol and then imported into the cilium (49). Remarkably, this is consistent with our finding that SDOS localizes to the rough ER and associates with actively translating ribosomes to control synthesis of constituent proteins of the primary cilium. In addition, our iCLIP and ribosome profiling analyses indicated an enrichment in transcripts involved in metabolic pathways. Intriguingly, both cilia defects (50) and metabolic dysfunction (51) have already been linked to cancer. In this view, the interaction between SDOS and the molecular chaperone TRAP1 at the ER, where they regulate protein synthesis in a likely cooperative manner, could contribute to the gene expression changes required by the metabolic rewiring in cancer cells, where TRAP1 plays a pivotal role in determining metabolism-driven cancer development and progression (52–55).

In conclusion, our study shows new functions and physiological roles of a newly identified RBP SDOS in regulating cilia formation and suggests potential uncharacterized links with novel developmental and degenerative diseases. Future studies in cancers and cilia-related congenital disorders are required to elucidate potential clinical relevance of the promising novel biomarker SDOS, and SDOS/TRAP1 cooperation, in these pathological conditions.

## DATA AVAILABILITY

iCLIP-Seq, Microarray and Ribosome profiling-Seq data is publicly available at the Gene Expression Omnibus (GEO) database under ID GSE118050.

## Supplementary Material

Supplementary Data
