# Editor’s Note on ‘Protein Syndesmos is a novel RNA-binding protein that regulates primary cilia formation’

**DOI:** 10.1093/nar/gkag828

**Published:** 2026-08-17

**Authors:** Julian E Sale, Barry L Stoddard

This an editor’s note regarding: Rosario Avolio, Aino I Järvelin, Shabaz Mohammed, Ilenia Agliarulo, Valentina Condelli, Pietro Zoppoli, Giovanni Calice, Daniela Sarnataro, Elias Bechara, Gian G Tartaglia, Matteo Landriscina, Alfredo Castello, Franca Esposito, Danilo S Matassa, Protein Syndesmos is a novel RNA-binding protein that regulates primary cilia formation, Nucleic Acids Research, Volume 46, Issue 22, 14 December 2018, Pages 12067–12 086, https://doi.org/10.1093/nar/gky873

In December 2025, the Editors were alerted to concerns about the apparent duplication of the SDOS blots in Figures 3B and 7C. These concerns have also been raised on PubPeer: https://pubpeer.com/publications/D73FB3EC736CC7D0511ACE34F083B4#1

The Editors subsequently investigated and contacted the corresponding authors.

The authors provided scanned films recovered from experiments performed in 2017 and explained their relationship to the published figures.

For Figure 3B, the original films used to assemble the published panel could not be located. However, the authors recovered contemporaneous replicate experiments showing puromycin incorporation assays in HeLa cells with SDOS knockdown or SDOS overexpression, together with the corresponding ACTIN and SDOS controls.

For Figure 7C, the authors recovered some, but not all, of the original films. Original films corresponding to the TMEM107, TMEM67, ACTIN, and SDOS panels were located, whereas the original films for the KIF7 and AHI1 panels could not be found. The authors also identified that the recovered SDOS blot was not the exact panel used in the published figure. They attributed this to an inadvertent figure-assembly error and stated that it does not affect the interpretation of the experiment or the study’s conclusions, noting that the SDOS blot served as a routine control for SDOS silencing.

The recovered original data and replicate experiments are available in the Supplementary Data.

The journal is publishing this note to document the availability of the underlying source material and the authors’ explanation regarding the preparation of these figures. While these issues may not affect the results, discussion, or conclusions presented in the article, in the absence of original data, the Editors advise readers to examine Figs. 3B and 7C with care.

## Supplementary Material

gkag828_Supplemental_File

