# Supplementary material for: Protein Syndesmos is a novel RNA-binding protein that regulates primary cilia formation
Source: Nucleic Acids Res. 2018 Sep 27;46(22):12067–86. doi: 10.1093/nar/gky873 (PMC6294507; doi:10.1093/nar/gky873)
Supplement: Supplementary Data [file gky873_supplemental_files.zip › Supplementary Materials and Methods.pdf]

Supplementary Materials and Methods

List of Oligos

Oligo name	Oligo sequence (5'-3')
ACKR3 FW	TGTGGGTTACAAAGCTGCCA
ACKR3 RV	GCGGGCAATCAAATGACCTCC
ACTB FW	CCTCACCCTGAAGTACCCCA
ACTB RV	TCGTCCCAGTTGGTGACGAT
AHI1 FW	GTGTCAACCTGGGGCTGAAT
AHI1 RV	ATGCAGAGGACTGAGAATGCAA
CASP1 FW	GCCTGTTCCTGTGATGTGGA
CASP1 RV	CACTTCCTGCCCACAGACAT
CC2D2A FW	AGGGGCATCTCCAACACTCA
CC2D2A RV	TCATTTTGGCTGGGATGGGT
CD83 FW	TTAATGGCCGGCTGGAAATG
CD83 RV	ATTAGCCCATGCAACAGCCT
DTNA FW	TTGGAATGTTCTCTGTGAACCAC
DTNA RV	TGAACAGCACCCTCAAAACA
ELFN2 FW	GGCTGGGAAGGAGAGGACTT
ELFN2 RV	GGAAGAGACCCATCTGCACC
FAM129A FW	TGGTTTCCAGATTGGAGCGA
FAM129A RV	CCAAAAACCATGTCTCGAGGT
KIF7 FW	ATGGAAACCTGACTGCCTGG
KIF7 RV	CAGTGGTCGAACTCGCAGG
LUC FW	TACAACACCCCAACATCTTCGA
LUC RV	GGAAGTTCACCGGCGTCAT
MACROD1 FW	GTGTTTGGCTACCCCTGTGA
MACROD1 RV	CTCGAGGAACACGCAGATGA
MAMDC2 FW	TGAATGTCCAAGGGTAAGTCAGT
MAMDC2 RV	AATTTCTATCCATGTGCTCATAGTG
NPHP1 FW	TTGGAGCAGCCTGATGTGAT
NPHP1 RV	CTCTTCTTCTGCCCACCTGAA
NTS FW	GGCTTTAGCTTGGAAGCAATGTT
NTS RV	TCATACAGCTGCCGTTTCAGA
OPN3 FW	AAAGAAACTGGCCAAAATGTGCT
OPN3 RV	TTGGAGTGACCAGGTGACCAT
PCK2 FW	CACCACTCAGCTGTTCTCCC
PCK2 RV	TTTGTGCACACGTCTCTCCA
RNF182 FW	AGGGGGCATTACTAAGACGAC
RNF182 RV	TGGGCATACATATTCCCGAAGT
RPGRIP1L FW	CATTTCCCAGGAGGCTACGG
RPGRIP1L RV	GTTGGACCAGACATGGCCTA
TCTN1 FW	GCACAACTGAGCAAGACTGC
TCTN1 RV	AAAGGGGCCACGTAATCTGG
TMEM107 FW	CCCCGAGGAGTATGACAAGC
TMEM107 RV	GCACTACAGTGAGCCCCAAT

TMEM67 FW	TGCCCAGAAAACATGAAAGGTG
TMEM67 RV	TCACAGAGCTCACAAGTTGCT
TOPORS FW	CGACACCGACCTAGCTTTCT
TOPORS RV	GCTAGTGCCAGCTTTAGGTGA
TTC21B FW	GGCGAGACAATCCCCAGAG
TTC21B RV	CCTGAAGACTGGATCACTTCCA
ZNF423 FW	CATGGATGCATAAGAAGAGGGTTGA
ZNF423 RV	TGGTTTTCTGATCGCACTCTGG
ZP3 FW	CCACGGTGTTCTCAGAGGAG
ZP3 RV	GTCCACAAACAACCGCAGTG
ZXDB FW	AGGCTTTGGCTAAATCTGACA
ZXDB RV	ATGGGGATGTGCAGGAAAGG

Differential gene expression analysis

Raw signal intensity data from Illumina HumanHT-12_V4_0_R2 microarrays was normalized, batch effect removed, and low-quality annotation probes excluded. Differentially expressed genes (p-value <0.05) were obtained using a moderated t-test on the linear model fit of the microarray data. All the steps were performed according to the microarray analysis best practice using R well known packages (R Core Team 2017; Ritchie et al. 2015). Differentially expressed genes (DEGs) between eGFP and SDOS-eGFP expressing HeLa cells were selected with differential score (DiffScore) cutoff set at ±13 (P<0.05). Downstream pathway analyses were performed only taking into account DEG with a Fold Change > 1.5. This data is available in the Gene Expression Omnibus (GEO) with ID GSE118050.

Bioinformatic analysis of iCLIP datasets

We used the demultiplex script from the iCount pipeline (http://icount.readthedocs.io/en/latest/) to trim off adapter sequences, to extract sample and molecular barcodes, and to demultiplex. We then mapped the reads to the human genome and annotation version GRCh38 downloaded from ENSEMBL using splice-aware mode of the STAR aligner (Dobin et al. 2013) though the iCount script mapstar. We then removed the PCR duplicates and identified the crosslinked sites on RNA using xlsites and peaks from the iCount pipeline. The crosslinked base is identified as the last base of the cDNA and first base of the read after the barcodes in this protocol. We then defined binding regions on RNA using a window of 10nt with BEDTools (Quinlan and Hall 2010). Binding regions were then assigned to genes and transcript features based on their overlap with annotated transcript features (ENSEMBL version GRCh38) utilising R/Bioconductor package GenomicRanges (Lawrence et al. 2013). We used the following assignment preference rule to assign those peaks that overlapped multiple annotated features at a gene level: protein coding exonic > miRNA > snRNA > snoRNA > rRNA > lincRNA > antisenseGenes > protein coding intronic > other > senseIntronic. Peaks overlapping multiple annotated exonic protein coding features were further assigned to transcript features according to the following assignment preference rule: CDS > 5UTR > 3UTR > other. In order to define reliable target genes for downstream analyses and experimental validation, we selected binding region with a False Discovery Rate < 0.05 and for which there was no signal detected for the gene in the negative control (eGFP) sample.

To better understand SDOS RNA-binding preferences, we used DREME (Bailey 2011) (MEME Suite version 5.0.1) to search for enriched sequence motifs around the SDOS

binding sites. Specifically, we selected the top 10% (448) of SDOS binding sites ranked by score value, and scanned +/-12 bp and +/-25 bp sequence regions around these positions. Randomly shuffled input sequence was used as a background in the search. We also used the same set of sequences to search for any potential repetitive motifs with gaps using GLAM2 (Frith et al. 2008).
The data is publicly available at the Gene Expression Omnibus (GEO) database under ID GSE118050.

Bioinformatic analysis of Ribosome Profiling datasets

Raw sequence reads were demultiplexed and adapter remnants were trimmed using cutadapt (Martin 2011). Reads derived from rRNAs were filtered out after a first pass mapping using bowtie2 and a custom composite rRNA genome. Remaining reads were aligned with Tophat2 on the hg38/GRCh38 human genome and the corresponding ENSEMBL transcriptome. Number of reads mapping "exon" was calculated with htseq-count (Anders et al. 2015) according to ENSEMBL annotation. Differentially expressed Ribosome Protected Fragments (RPFs) were calculated using the DEseq2 bioconductor R package (Love et al. 2014). For downstream analysis, we considered only RPFs with Fold Change > 3.0. Selected RPFs were normalized to the Microarray results in order to select only those genes regulated at translational level.
The data is publicly available at the Gene Expression Omnibus (GEO) database under ID GSE118050.

Gene Ontology Analyses

iCLIP and RP data (significantly impacted pathways, biological processes, cellular component, diseases) were analysed using Advaita Bio's iPathwayGuide (http://www.advaitabio.com/ipathwayguide). To highlight a possible activation/deactivation of biological functions and pathways of microarray data, we perfrmed the gene set enrichment analysis (GSEA) (Subramanian et al. 2005) of differentially expressed genes.

References

- Anders S, Pyl PT, Huber W. HTSeq--a Python framework to work with high-throughput sequencing data. Bioinformatics. 2015;31(2):166-9.
- Bailey TL. DREME: motif discovery in transcription factor ChIP-seq data. Bioinformatics. 2011;27(12):1653-9.
- Dobin A, Davis CA, Schlesinger F, Drenkow J, Zaleski C, Jha S, Batut P, Chaisson M, Gingeras TR. STAR: ultrafast universal RNA-seq aligner. Bioinformatics. 2013;29(1):15-21.
- Frith MC, Saunders NF, Kobe B, Bailey TL. Discovering sequence motifs with arbitrary insertions and deletions. PLoS Comput Biol. 2008;4(4):e1000071.
- Lawrence M, Huber W, Pagès H, Aboyoun P, Carlson M, Gentleman R, Morgan MT, Carey VJ. Software for computing and annotating genomic ranges. PLoS Comput Biol. 2013;9(8):e1003118.
- Love MI, Huber W and Anders S. Moderated estimation of fold change and dispersion for RNA-seq data with DESeq2. Genome Biology 2014; 15: 550.
- Martin M. Cutadapt removes adapter sequences from high-throughput sequencing reads. EMBnet.journal 2011;17(1): 10-12.

- Quinlan AR, Hall IM.BEDTools: a flexible suite of utilities for comparing genomic features. Bioinformatics. 2010;26(6):841-2.
- Ritchie ME, Phipson B, Wu D, Hu Y, Law CW, Shi W, Smyth GK. limma powers differential expression analyses for RNA-sequencing and microarray studies. Nucleic Acids Res. 2015;43(7):e47.
- Subramanian A, Tamayo P, Mootha VK, Mukherjee S, Ebert BL, Gillette MA, Paulovich A, Pomeroy SL, Golub TR, Lander ES, Mesirov JP. Gene set enrichment analysis: a knowledge-based approach for interpreting genome-wide expression profiles. Proc Natl Acad Sci U S A. 2005;102(43):15545-50.
