# Supplementary material for: Protein Syndesmos is a novel RNA-binding protein that regulates primary cilia formation
Source: Nucleic Acids Res. 2018 Sep 27;46(22):12067–86. doi: 10.1093/nar/gky873 (PMC6294507; doi:10.1093/nar/gky873)
Supplement: Supplementary Data [file gky873_supplemental_files.zip › Supplementary Table 1.pdf]

**Supplementary Table 1:** Amino acids present in the electrostatic patches at the surface of SDOS and NUDT16 according to BindUP predictions. The PDB IDs 2xsq for NUDT16 and 3kvh for SDOS were used for the prediction.

| Name | Amino acids |
|---|---|
| SDOS positive patch 1 | LEU9 LYS10 GLN11 ILE12 GLU16 ARG19 LEU20 GLY21 GLY23 TRP24 LEU97 SER98 HIS100 LEU101 THR102 ARG107 ASN163 ALA164 PHE165 VAL166 SER167 THR168 LYS170 CYS171 GLN172 LEU174 PHE175 LYS178 VAL179 GLU185 LEU188 LEU192 ALA193 THR196 GLU197 GLN199 |
| SDOS positive patch 2 | GLN39 PHE41 GLY42 ARG43 PRO45 MET46 ARG47 PHE48 LEU147 TYR148 THR149 GLN150 LYS151 ASP152 ARG153 PHE157 PRO158 ASN181 MET182 MET183 PRO184 GLU185 GLU186 LYS187 ALA191 ALA194 |
| NUDT16 positive patch 1 | ALA4 ARG5 TRP19 HIS24 HIS99 VAL100 GLY101 SER102 VAL106 ILE164 GLY165 SER166 ALA167 |
| NUDT16 positive patch 2 | ASP71 ASN74 ARG75 ARG78 ALA86 ARG89 |
