# Supplementary material for: Editor’s Note on ‘Protein Syndesmos is a novel RNA-binding protein that regulates primary cilia formation’
Source: Nucleic Acids Res. 2026 Aug 17;54(15):gkag828. doi: 10.1093/nar/gkag828 (PMC13478743; doi:10.1093/nar/gkag828)
Supplement: Supplementary file 1 [file gkag828_supplemental_file.pdf]

Same anti-puromycin experiments, different exposures

Two contemporaneous repeat experiments

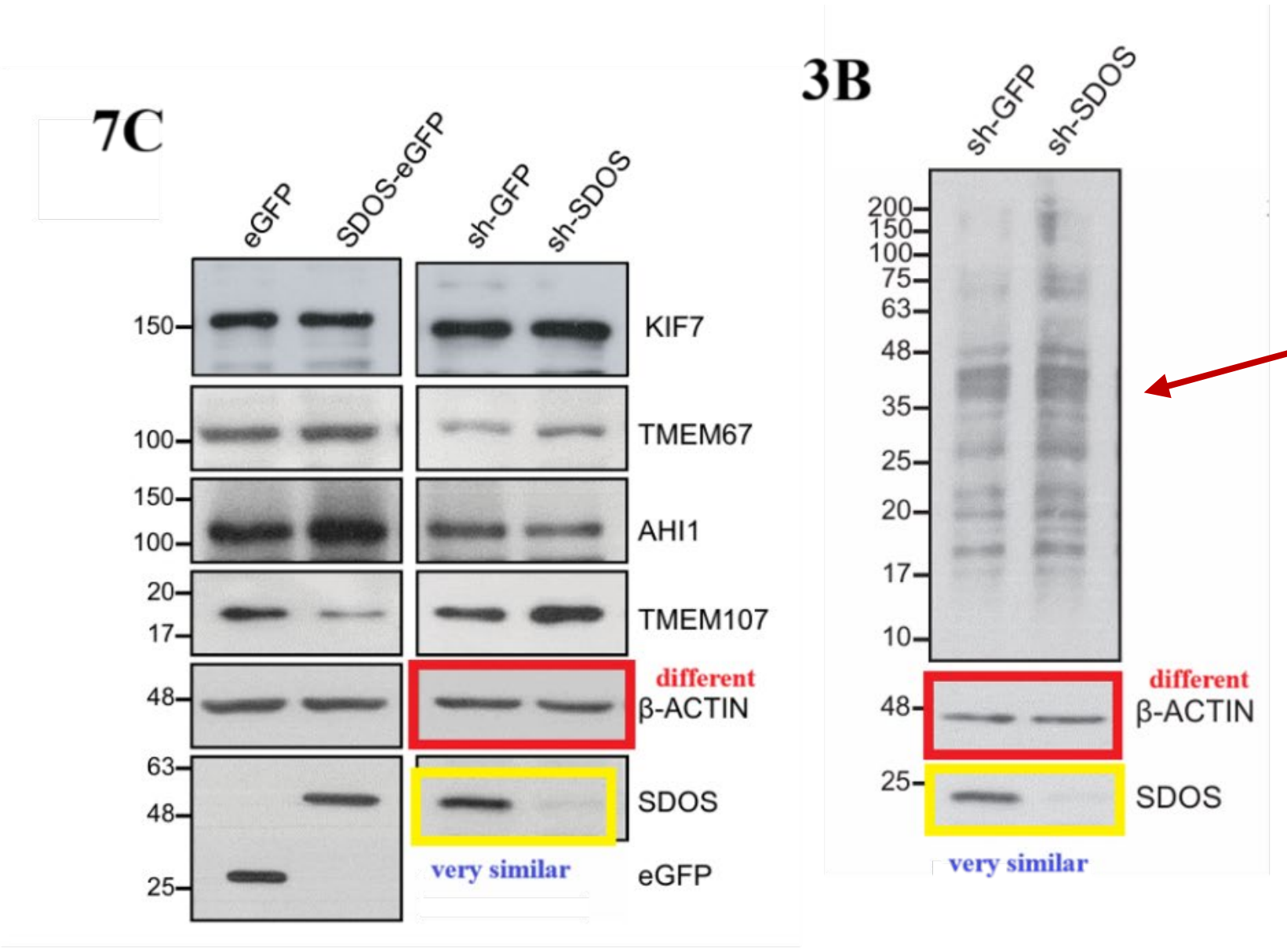

Nucleic Acids Res . 2018 Dec 14;46(22):12067-12086.

Actin normalization

Anti-SDOS hybridization, showing SDOS silencing in lane 2 versus lane 1 and in lane 4 versus lane 3, as well as SDOS-GFP expression in lane 6 (indicated by arrows on the right)

High exposure following anti-ACTIN hybridization, showing residual anti-puromycin signal from the first hybridization

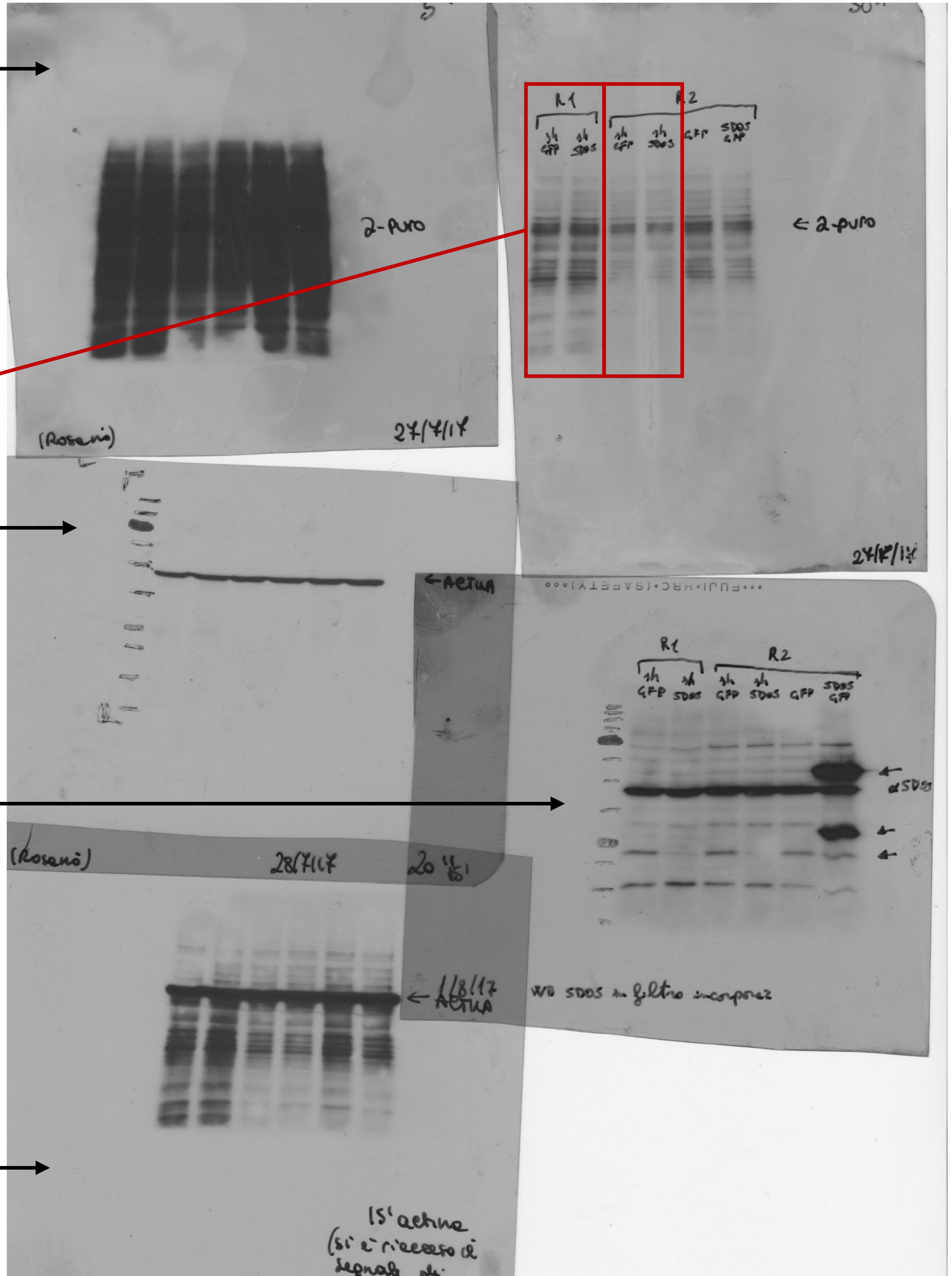

Actin normalization

anti-TMEM107 hybridization. The first two lanes, corresponding to those used in Figure 7C, are marked with an asterisk

Green boxes: data not available

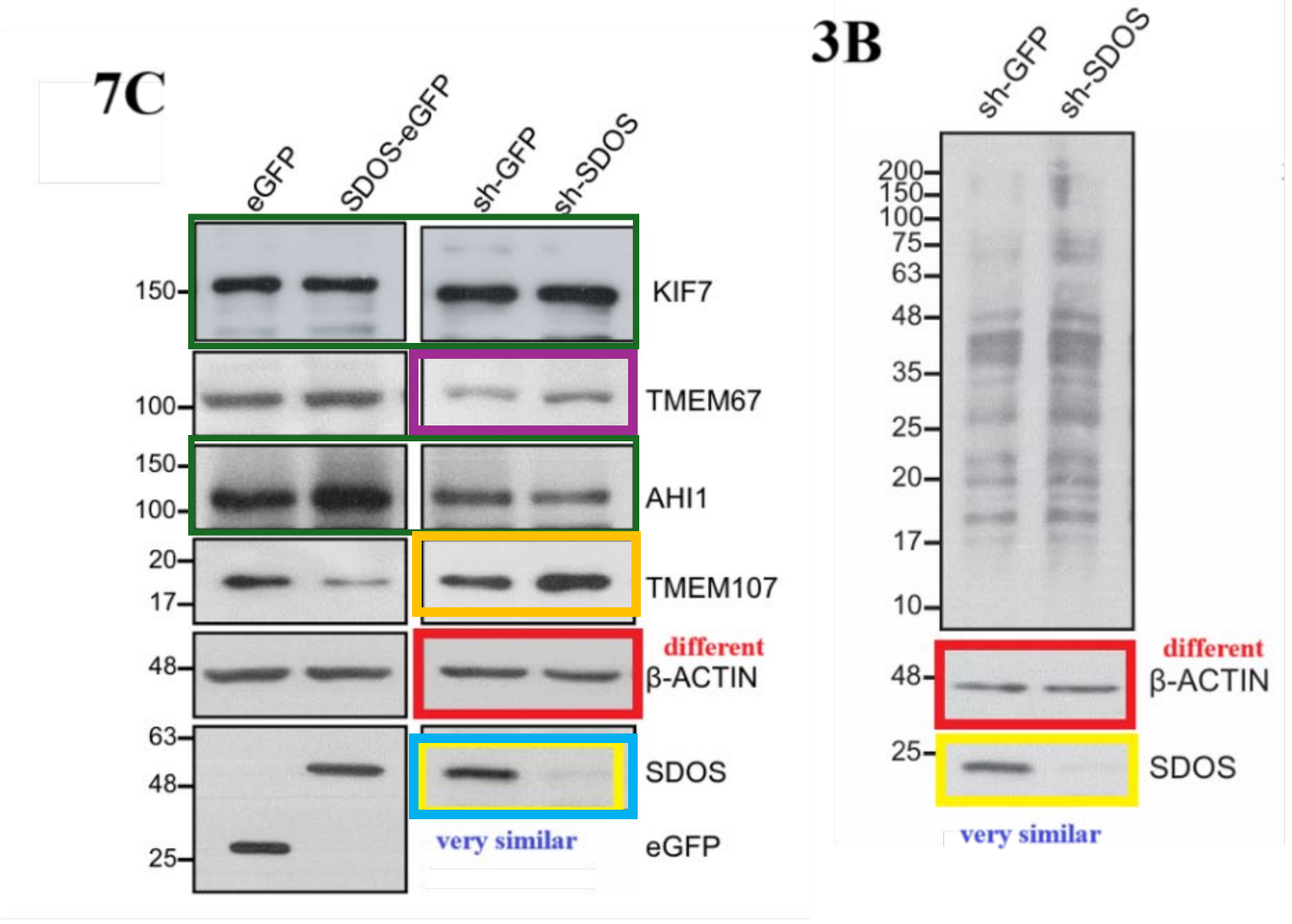

Nucleic Acids Res . 2018 Dec 14;46(22):12067-12086.

TMEM67 panel. Again, the relevant bands are marked with an asterisk

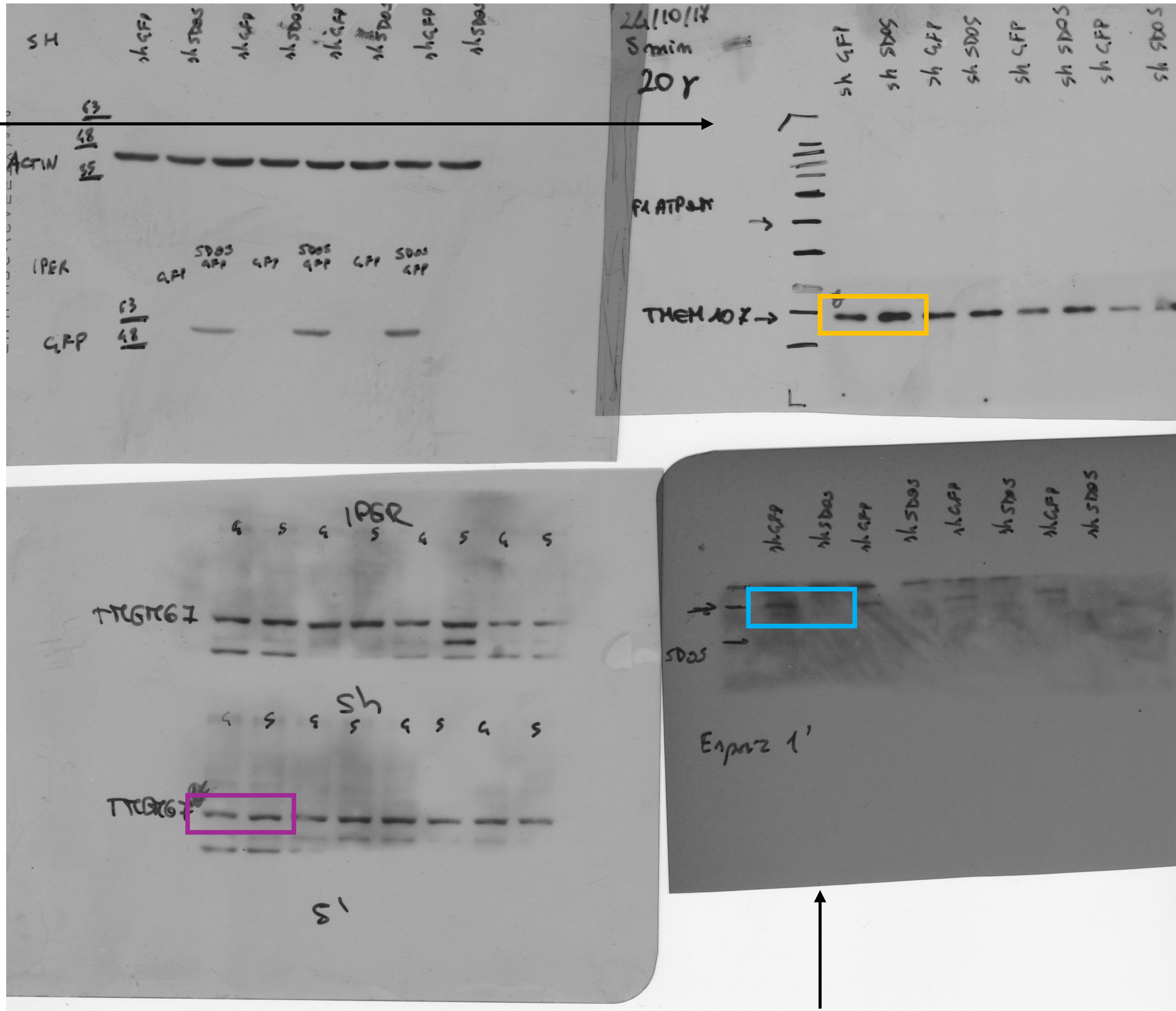

SDOS hybridization (indicated by an arrow on the left), which is the panel that raised the concern
